# An Investigation into the Planning of Cutting Interpolation Point Positions to Improve the Surface Quality of Satellite Laser Communication Reflectors

**DOI:** 10.3390/mi17080938

**Published:** 2026-08-06

**Authors:** Guilin Zhuang, Qian Yu, Zihao Zeng

**Affiliations:** State Key Laboratory of Chips and Systems for Advanced Light Field Display, School of Mechanical Engineering, Beijing Institute of Technology, Beijing 100081, China

**Keywords:** spherical surface, reflectivity, vibration, interpolation point planning algorithm

## Abstract

Spherical/aspherical mirrors are widely used in satellite communication and imaging systems, but their reflectivity is affected by the surface roughness value. The vibration of the machine tool system is one of the most important factors affecting the surface roughness. This paper systematically suppresses vibration through different ways, reduces the peak and valley value of workpiece surface roughness, and improves the specular reflectivity (i.e., zero-order diffraction efficiency) of the machined surface. This paper establishes a reflectance model for machined surfaces considering surface aberration using rigorous coupled wave theory. Based on this model, the relationship between reflectivity and processed surface morphology was calculated. According to the influence of different vibration modes on surface morphology, the influence of different vibration morphology on reflectivity is studied. In order to reduce cutting vibration, a new adaptive interpolation point planning algorithm has been innovatively proposed for planning the position of each interpolation point on the meridian of the workpiece. The specular reflectivity of uncoated bare spherical/aspherical mirror surfaces processed by adaptive interpolation point planning algorithm can reach over 90%, providing a high-quality substrate for subsequent high-reflection optical coatings.

## 1. Introduction

The modeling of optical diffraction effect is mainly divided into two types: scalar diffraction modeling and vector diffraction modeling. Commonly used vector diffraction theory analysis methods such as finite element method, boundary element method, and rigorous coupled wave analysis theory, coupled wave theory [1,2,3], and modal theory [4,5,6] are widely used because of their high calculation accuracy and ease of programming.

At present, there are many technological methods to improve the reflectivity of optical lenses. Grosjean et al. [7] improved solar reflectivity by using a dielectric layer, replaced silver dielectric layer with aluminum- and magnesium fluoride-mixed dielectric layer in the solar reflector, and adopted aperiodic stacked thin film coating technology. In theory, the solar reflectivity reached 96.1%, which was higher than that of silver’s 95.5%. Larruquert et al. [8] obtained a new method to enhance the reflectivity based on the multilayer superposition of various materials with different absorptivity. This method was developed for materials with small refractive index difference, and in some cases it is also effective for materials with large refractive index difference, which can effectively improve the surface reflectivity in a very wide wavelength band and a large incident angle range.

There is an obvious correspondence between diffraction effect and surface roughness. At present, the main method to reduce the surface roughness of optical mirrors is polishing. In addition, ultra-precision turning, ultra-precision ironing technology and Haokeneng technology can also reduce the surface roughness of the workpiece. Peng et al. [9] proposed a new combined process method combining Magneto Rheological Finishing (MRF) and Hydrodynamic Effect Polishing (HEP) technology to process the surface of quartz glass to RMS 0.407 nm. Venkata et al. [10] studied the quantitative relationship between the surface roughness and integral scattering of ultra-precision polishing. The results show that the smaller the surface roughness RMS value, the lower the influence of integral scattering on reflectivity.

Corning Company of the United States uses LEC technology [11] to obtain a no tool mark surface, which reduces the diffraction effect of the mirror surface. This technology is successfully applied to the manufacturing process of the space telescope mirror. At present, this technology is still kept secret, but Corning Company has put forward several ways to reduce the surface roughness: reducing the grain size of workpiece materials, controlling external vibration, improving the quality of diamond cutting tools, reasonably selecting cutting fluid types, optimizing interpolation algorithm and clamping method of workpiece, etc.

At present, the ultra-precision ironing technology is deeply studied in Jilin University and Changchun University of Science and Technology. Wang et al. [12] and Lu et al. [13] have studied the influence of two-dimensional and three-dimensional elliptical vibration-assisted ironing technology on surface roughness. Although the ultra-precision ironing method has a good development prospect, according to the existing literature, its machining accuracy is far lower than that of polishing and ultra-precision turning (Ra37.7 nm).

To sum up, there are several methods to reduce diffraction efficiency and improve reflectivity, such as reducing surface roughness, coating, ironing and replication. For the aluminum alloy reflector used in this subject and the applied near ultraviolet, visible and near-infrared bands, reducing the surface roughness is the most direct and effective means.

In this paper, a reflectivity model considering aberration is established, and through analysis, an appropriate spot diameter is selected to avoid the influence of aberration on reflectivity. Combined with the practical application of mirrors, because the incident angles of light rays are different at different positions of spherical/aspherical mirrors, this paper considers the influence of shadowing effect on reflectivity, and explores the influence degree of shadowing effect on reflectivity under different surface topography. In order to suppress the influence of vibration on the surface topography, a new interpolation point planning method is proposed in this paper. The experimental results show the effectiveness of the vibration suppression method. The interpolation point planning method proposed in this paper can lay a theoretical foundation for ultra-precision turning of spherical/aspheric surfaces with high surface quality. Unless otherwise specified, the reflectivity referred to in this paper is the specular reflectivity (i.e., zero-order diffraction efficiency) of mirror light, that is, the ratio of the zero-order reflected light intensity to the incident light intensity.

## 2. Theoretical Modeling

### 2.1. Dynamic Response Analysis of Multi-Body Systems

**Figure 1:**
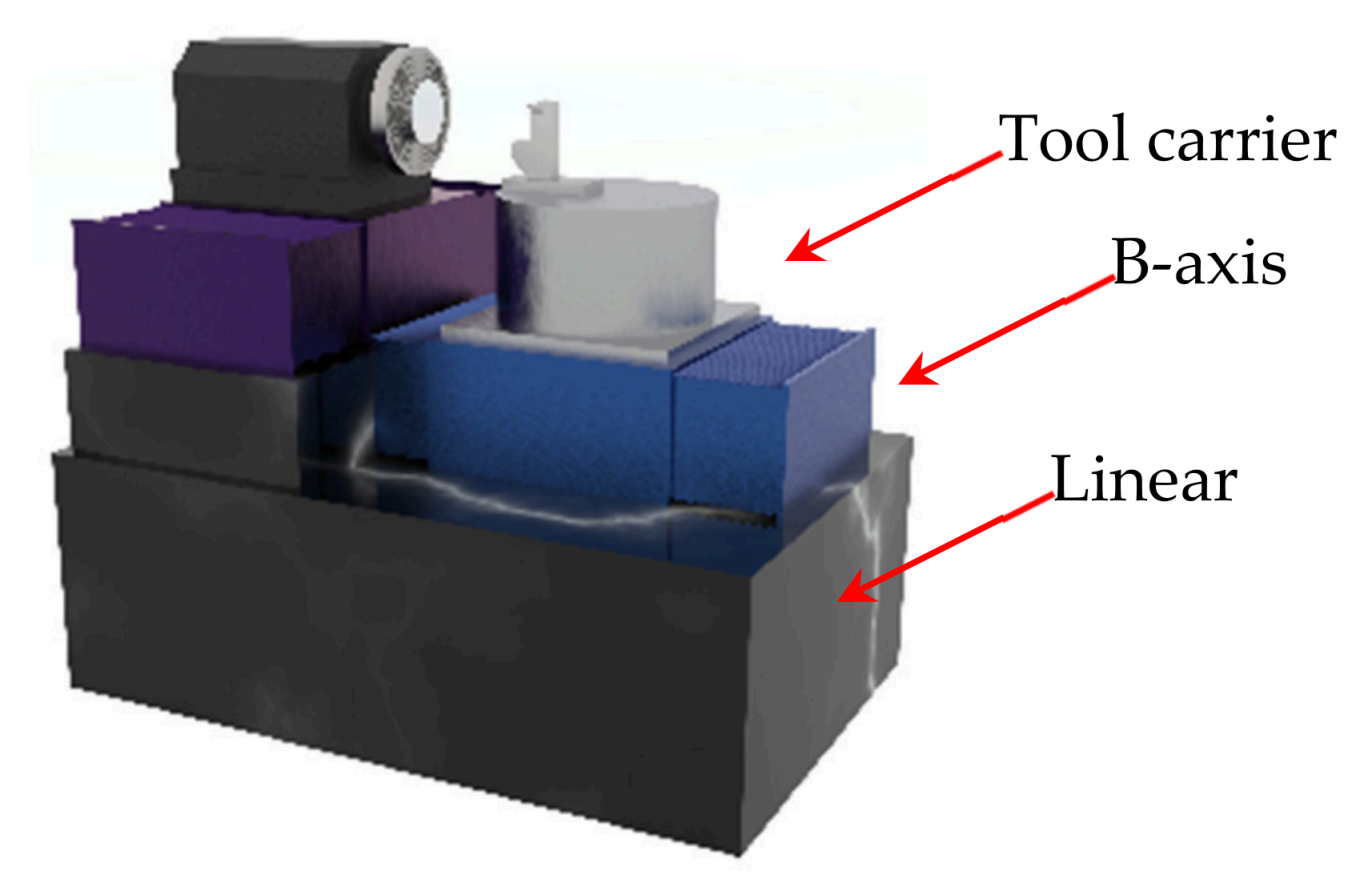
Schematic diagram of machine tool structure.

In diamond turning, vibration is one of the most important factors affecting the machined surface roughness. To simplify the analysis, the study categorizes machine tools into two distinct multi-rigid-body systems along orthogonal axes: Z-direction and X-direction (as shown in Figure 1). The Z-axis configuration comprises three primary components: the linear guide assembly, B-axis rotary unit, and tool carrier. Separate dynamic equations are formulated for each subsystem (guideway, B-axis, and tool holder) to describe their mechanical interactions.

The fundamental dynamic equation governing these three interconnected elements can be represented as

(1) $$\left\{\begin{array}{l} M_{n}v_{i,tt}+C_{n}v_{i,t}+K_{n}v_{i}=f_{i}\\ J_{n}v_{\theta i,tt}+C_{\theta n}v_{\theta i,t}+K_{\theta n}v_{\theta i}=T_{qi} \end{array}\right.$$

where $M_{n}$ represents the inertial parameter matrix quantifying mass distribution across three body components; *J_n_* denotes rotational inertia properties; $v_{i}$ is the displacement matrix, which characterizes the motion states of three body elements; $v_{\theta i}$ is the rotation angle matrix. Temporal derivatives are indicated through subscript notations: *t* for velocity components and *tt* for acceleration terms. Structural resistance characteristics are defined by stiffness array: $K_{n}$, $K_{\theta n}$ and energy dissipation parameters through damping array: $C_{n}$, $C_{\theta n}$. External influences are mathematically represented by force vector *f_i_* and torque vector *T_qi_* acting on system components.

Regarding the Z-axis guide mechanism, its slide carriage is modeled as a six-degree-of-freedom rigid body, exhibiting spatial oscillations. This mechanical behavior is governed by the dynamic equations describing translational vibrations.

(2) $$\left\{\begin{array}{l} m_{1}\ddot{z}+\left(c_{gz}+C_{gz}\right)\dot{z}+\left(k_{gz}+K_{gz}\right)z=F_{oz}+F_{encoder}\\ m_{1}\ddot{y}+\left(c_{gy}+C_{gy}\right)\dot{y}+\left(k_{gy}+K_{ay}\right)y=G_{holder}+F_{oy}\\ m_{1}\ddot{x}+\left(c_{gx}+C_{gx}\right)\dot{x}+\left(k_{gx}+K_{gx}\right)x=F_{ox} \end{array}\right.$$

where *C_gn_* (*n* = *x*, *y*, *z*) corresponds to the damping coefficient in the coupling interface connecting the B-axis and guideway slide carriage, with calculation methods detailed in prior research [14]; *K_gn_* (*n* = *x*, *y*, *z*) quantifies the stiffness characteristics within this mechanical interface; *F_on_* (*n* = *x*, *y*, *z*) characterizes the triaxial electromagnetic forces generated during operation; *G_holder_* accounts for gravitational effects on the tool-holding assembly; *F_encoder_* describes thrust variations in linear motors caused by positional sensing errors, as documented in reference [14]. *k_gn_* and *c_gn_* (*n* = *x*, *y*, *z*) respectively define the lubricating oil film’s stiffness and damping properties, parameters which were thoroughly investigated and modeled in earlier scholarly work [15].

Considering the structural arrangement of the tool holder and B-axis along the guideway, the center of mass and the motion center of the sliding carriage are not aligned, with positional deviations defined as *e_x_* and *e_y_* along the X- and Y-axis respectively. Since the *e_z_* component does not generate angular momentum in the Z-axis orientation, its contribution is considered negligible. Consequently, accounting for angular momentum effects, the rotational vibration dynamics governing the Z-axis guideway can be formulated

(3) $$\left\{\begin{array}{l} J_{x1}\ddot{\theta }-m_{1}e_{x}e_{y}\ddot{\phi }+\left(k_{\theta x}+K_{\theta x}\right)\theta +\left(c_{\theta x}+C_{\theta x}\right)\dot{\theta }=G_{holder}\cdot l_{x}+k_{y}\cdot l_{2}^{2}\cdot \theta +K_{y}\cdot l_{2}^{\prime 2}\cdot \theta +F_{oz}\cdot d\\ J_{y1}\ddot{\phi }-m_{1}e_{y}e_{x}\ddot{\theta }+\left(k_{\theta y}+K_{\theta y}\right)\phi +\left(c_{\theta y}+C_{\theta y}\right)\dot{\phi }=k_{x}\cdot l_{1}^{2}\cdot \phi +K_{x}\cdot l_{1}^{\prime 2}\cdot \phi \\ J_{z1}\ddot{\gamma }+\left(k_{\theta z}+K_{\theta z}\right)\gamma +\left(c_{\theta z}+C_{\theta z}\right)\dot{\gamma }=G_{holder}\cdot l_{z}+k_{y}\cdot l_{2}^{2}\cdot \gamma +k_{z}\cdot l_{3}^{2}\cdot \gamma +K_{y}\cdot l_{2}^{\prime 2}\cdot \gamma +K_{z}\cdot l_{3}^{\prime 2}\cdot \gamma +F_{ox}\cdot d \end{array}\right.$$

where *J_n_*_1_(*n* = *x*, *y*, *z*) denotes the guideway’s moment of inertia across various axes; $k_{\theta n}$ (*n* = *x*, *y*, *z*) represents the rotational stiffness provided by the guideway’s oil film; $c_{\theta n}$ (*n* = *x*, *y*, *z*) corresponds to the rotational damping characteristics of the oil film; $C_{\theta n}$ (*n* = *x*, *y*, *z*) indicates the rotational damping present in the B-axis–guideway coupling; $K_{\theta n}$ (*n* = *x*, *y*, *z*) signifies the rotational stiffness within the B-axis and guideway slide carriage connection. The mass center offsets *l_x_* and *l_z_* along the X- and Z-axes result from positional deviations of both the B-axis and tool holder on the slide carriage; $l_{n}^{\prime }$ (*n* = 1, 2, 3) quantifies the spatial separation between bolt stiffness equivalent points and the guideway slide carriage’s gravitational center; *l_n_* (*n* = 1, 2, 3) specifies the dimensional interval between oil film stiffness application points and the slide carriage’s mass centroid. These geometric relationships fundamentally influence the system’s dynamic behavior through their spatial configurations relative to critical components; *d* represents the separation between the linear motor’s mass center and that of the slide carriage; *θ*, *ϕ* and *γ* are the rotational angles around the X-, Y-, and Z-axes, respectively.

The dynamic equations governing the tool holder’s translational and rotational vibrations are expressed as

(4) $$\left\{\begin{array}{l} m_{3}{\ddot{z}}_{1}+\left(C_{tz}\right){\dot{z}}_{1}+\left(K_{tz}\right)z_{1}=F_{z}\\ m_{3}{\ddot{y}}_{1}+\left(C_{ty}\right){\dot{y}}_{1}+\left(K_{ty}\right)y_{1}=F_{y}\\ m_{3}{\ddot{x}}_{1}+\left(C_{tx}\right){\dot{x}}_{1}+\left(K_{tx}\right)x_{1}=F_{x} \end{array}\right.$$

(5) $$\left\{\begin{array}{l} J_{x3}{\ddot{\theta }}_{1}+\left(K_{t\theta x}\right)\theta_{1}+\left(C_{t\theta x}\right){\dot{\theta }}_{1}=-K_{ty}\cdot l_{2}^{\prime \prime 2}\cdot \theta_{1}+F_{y}\cdot d_{2}-F_{z}\cdot d_{3}\\ J_{y3}{\ddot{\phi }}_{1}+\left(K_{t\theta y}\right)\phi_{1}+\left(C_{t\theta y}\right){\dot{\phi }}_{1}=-K_{tx}\cdot l_{1}^{\prime \prime 2}\cdot \phi_{1}+F_{z}\cdot d_{3}-F_{x}\cdot d_{1}\\ J_{z3}{\ddot{\gamma }}_{1}+\left(K_{t\theta z}\right)\gamma_{1}+\left(C_{t\theta z}\right){\dot{\gamma }}_{1}=-K_{ty}\cdot l_{2}^{\prime \prime 2}\cdot \gamma_{1}+K_{tz}\cdot l_{3}^{\prime \prime 2}\cdot \gamma_{1}-F_{y}\cdot d_{2}+F_{x}\cdot d_{1} \end{array}\right.$$

where *K_tn_* and *C_tn_*(*n* = *x*, *y*, *z*) indicate the stiffness coefficients and damping properties of the B-axis-to-tool-holder bolted connection; $K_{t\theta n}$ and $C_{t\theta n}$ (*n*= *x*, *y*, *z*) represent rotational stiffness and damping characteristics in the same joint interface; *m_3_* is the tool holder mass; *d*_1_, *d*_2_ and *d*_3_ are the spatial offsets between the cutting force application point and the tool holder’s centroid along orthogonal axes; $l_{n}^{\prime \prime }$ (*n* = 1, 2, 3) quantifies the distance from bolt joint stiffness equivalent points to the tool holder’s center of mass; $\theta_{1},\;\phi_{1}$ and $\gamma_{1}$ describe rotational displacements about the three coordinate axes.

The dynamic equations governing the B-axis motion can be derived through analogous methodologies, expressed as

(6) $$\left\{\begin{array}{l} m_{2}{\ddot{z}}_{2}-\left(C_{gz}+C_{tz}\right){\dot{z}}_{2}-\left(K_{gz}+K_{tz}\right)z_{2}=0\\ m_{2}{\ddot{y}}_{2}-\left(C_{gy}+C_{ty}\right){\dot{y}}_{2}-\left(K_{gy}+K_{ty}\right)y_{2}=0\\ m_{2}{\ddot{x}}_{2}-\left(C_{gx}+C_{tx}\right){\dot{x}}_{2}-\left(K_{gx}+K_{tx}\right)x_{2}=0 \end{array}\right.$$

(7) $$\left\{\begin{array}{l} J_{x2}{\ddot{\theta }}_{2}-\left(K_{\theta x}+K_{t\theta x}\right)\theta_{2}-\left(C_{\theta x}+C_{t\theta x}\right){\dot{\theta }}_{2}=K_{y}\cdot l_{1}^{\prime 2}\cdot \theta_{2}-K_{y}\cdot l_{2}^{\prime 2}\cdot \theta \\ J_{y2}{\ddot{\phi }}_{2}-\left(K_{\theta y}+K_{t\theta y}\right)\phi_{2}-\left(C_{\theta y}+C_{t\theta y}\right){\dot{\phi }}_{2}=K_{x}\cdot l_{1}^{\prime 2}\cdot \phi_{1}-K_{x}\cdot l_{1}^{\prime 2}\cdot \phi \\ J_{z2}{\ddot{\gamma }}_{2}-\left(K_{\theta z}+K_{t\theta z}\right)\gamma_{2}-\left(C_{\theta z}+C_{t\theta z}\right){\dot{\gamma }}_{2}=K_{y}\cdot l_{2}^{\prime 2}\cdot \gamma_{1}+K_{x}\cdot l_{3}^{\prime 2}\cdot \gamma_{1}-K_{y}\cdot l_{2}^{\prime 2}\cdot \gamma +K_{z}\cdot l_{3}^{\prime 2}\cdot \gamma \end{array}\right.$$

where *m*_2_ represents the B-axis component’s mass; *J_n_*_2_(*n* = *x*, *y*, *z*) denotes its moment of inertia in respective directions; *x*_2_, *y*_2_ and *z*_2_ indicate translational displacements. The angular rotations about the X-, Y-, and Z-axes are respectively symbolized by $\theta_{2},\;\phi_{2}$ and $\gamma_{2}$.

By incorporating these analytical outcomes into Equation (2), the mechanical equation for the Z-axis multi-body system accounting for translational vibrations is formulated as

(8) $$M_{Z}\cdot v_{Ztt}+C_{Z}\cdot v_{Zt}+K_{Z}\cdot v_{Z}=f_{Z}$$

where *v_Z_* represents the displacement matrix; $v_{Ztt}$ corresponds to the acceleration matrix; $v_{Zt}$ signifies the velocity matrix. The mass matrix *M_Z_* ($M_{Z}=\left[\begin{array}{ccc} m_{1} & & \\ & m_{2} & \\ & & m_{3} \end{array}\right]$) incorporates the modal masses *m*_1_, *m*_2_, and *m*_3_ for the Z-direction guideway, B-axis, and tool holder components, respectively. The stiffness matrix *K_Z_* ($K_{Z}=\left[\begin{array}{ccc} K_{1} & & \\ & K_{2} & \\ & & K_{3} \end{array}\right]$) combines the structural rigidity parameters *K*_1_ (Z-direction guideway), *K*_2_ (B-axis), and *K*_3_ (tool holder). Damping characteristics are captured in matrix *C_Z_* ($C_{Z}=\left[\begin{array}{ccc} C_{1} & & \\ & C_{2} & \\ & & C_{3} \end{array}\right]$) through coefficients *C*_1_, *C*_2_ and *C*_3_ corresponding to each respective mechanical component. The external force matrix *f_Z_* ($f_{Z}={\left[\begin{array}{ccc} f_{1} & f_{2} & f_{3} \end{array}\right]}^{T}$) aggregates operational forces *f*_1_, *f*_2_ and *f*_3_ acting on the Z-direction guideway, B-axis assembly, and cutting tool holder.

Following the established methodology for Z-axis multi-body dynamics, equivalent dynamic equations can be derived for the X-direction motion system by applying analogous principles. However, detailed derivations for the X-direction system have been omitted in this discussion for conciseness.

### 2.2. Reflectance Modeling

**Figure 2:**
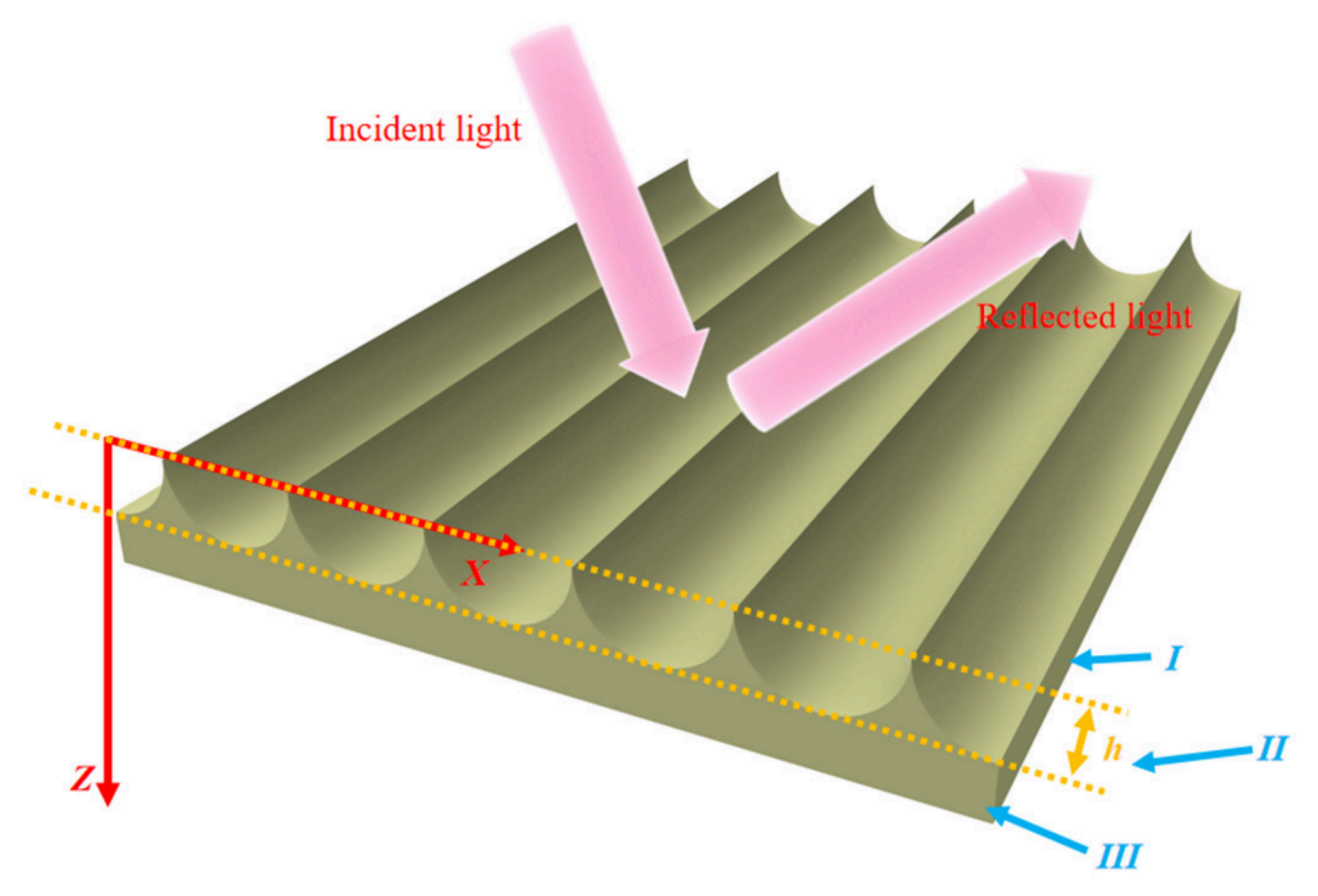
Schematic diagram of turning area division.

When the feature size is close to the wavelength of the incident light, the scalar diffraction theory and vector diffraction theory based on Green’s function are no longer applicable, and the vector rigorous coupled wave method of grating diffraction is needed. Figure 2 shows a three-dimensional sectional view of the ultra-precision turning surface, which is composed of two uniform media, incident media and grating media. The incident medium is air, and its dielectric constant and permeability are and $\mu_{0}$, respectively. According to the viewpoint of thin film optics, the surface can be divided into three areas: Area I is the incident dielectric layer, *z <* 0. Area II is a grating layer, 0 < *z* < *h*. Area III is the base, *z* > *h*.

For the calculation of the dielectric constant of grating layer, the periodic grating can be regarded as a periodic function $\varepsilon_{r}\left(x\right)$ with the change in relative dielectric constant $\varepsilon_{r}$ with x.

(9) $$\varepsilon_{r}\left(x\right)=\sum_{l=-\infty }^{+\infty }\left(\varepsilon_{r,l}+n_{0}\delta \left[l\right]\right)e^{-j\frac{2\pi l}{d}x}$$

where *n*_0_ is the refractive index; *l* represents the diffraction wave order; $\delta \left[l\right]$ represents the unit sequence; $\varepsilon_{r,l}$ is the coefficient of the Fourier series; *d* is the grating (tool mark) period.

The reflectivity can be obtained by substituting the above equation into Maxwell equations.

(10) $$\left\{\begin{array}{l} \nabla \times E\left(r,\omega \right)=j\omega \mu_{0}H\left(r,\omega \right)\\ \nabla \times H\left(r,\omega \right)=-j\omega \varepsilon_{r}\left(x\right)E\left(r,\omega \right) \end{array}\right.$$

where $E\left(r,\omega \right)$ and $H\left(r,\omega \right)$ are the electric field component and magnetic field component of diffracted light, respectively; $\mu_{0}$ and ω are the permeability and frequency of light, respectively. With the above method, the spatial distribution and reflectivity of diffracted light can be obtained. In this study, the RCWA simulations were computationally executed using VirtualLab Fusion to calculate the diffraction efficiencies.

### 2.3. Influence of Vector Aberration on Reflectivity

Through the research of Rayleigh et al. [16], it is found that the aberration of the system also affects the diffraction phenomenon, and the diffraction phenomenon affects the reflectivity of ultra-precision surfaces. In the context of this work, “aberration” refers to the wavefront distortion induced by macro-form errors and local curvature variations in the aspherical surface. This wavefront distortion causes the reflected light to diverge from the ideal focal point, thereby reducing the effective specular reflection intensity captured by a detector. The article specifically discusses the quantitative relationship between aberration and diffraction.

Firstly, the polar coordinate system is set as (ρ,θ). According to the aberration theory, this paper mainly studies three basic aberration components, namely: spherical aberration, coma and astigmatism. The expression of vector aberration *W* under normal incidence is

(11) $$W=W_{040}{\left(\vec{\rho }\cdot \vec{\rho }\right)}^{2}+W_{031}\left(\vec{\rho }\cdot \vec{\rho }\right)\left[\left(\vec{H}-{\vec{\sigma }}_{J}\right)\cdot \vec{\rho }\right]+\frac{1}{2}W_{022}{\left[\left(\vec{H}-{\vec{\sigma }}_{J}\right)\cdot \vec{\rho }\right]}^{2}$$

where *W_lmn_* represents the coefficient of aberration; λ is the wavelength; *l*, *m*, *n* are constants, which change with different forms of aberration. For example, for spherical aberration, *l* = 0, *m* = 4, *n* = 0. The first term of the above formula represents spherical aberration; the second term represents coma; the third term represents astigmatism; ${\vec{\sigma }}_{J}$ is the vector proposed by Buchroeder, which is represented as the offset vector in the vector image field. Specifically, this offset vector is explicitly derived from the macroscopic geometry of the workpiece, determined by the radius of curvature (for spherical surfaces) or conic constants (for aspherical surfaces) coupled with the specific incident angle; $\vec{H}$ represents the vector coordinates of any point in the image field; $\vec{\rho }$ is the vector radius of any point within the coordinate system.

**Figure 3:**
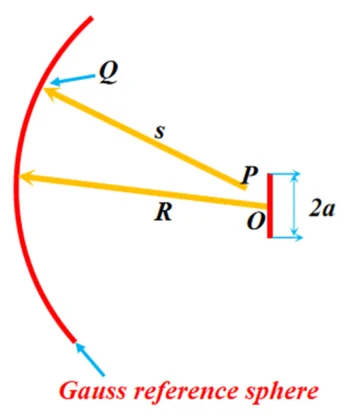
Gauss reference sphere.

As shown in Figure 3, let *R* represent the radius of the sphere. *s* is the distance between the point *Q* on the sphere and any point P in the image area. The field disturbance at the Q point is represented by $Ae^{ik\left(W-R\right)}$, where *A* is the amplitude of the light wave at the Q point.

Let $\left(\xi ,\eta ,\zeta \right)$ be the coordinates of point Q, (*x*, *y*, *z*) be the coordinates of point P, and let *a* be the radius of the exit pupil, that is, the radius of the light source. After integral calculation, we can get

(12) $$k\left(s-R\right)=-v\rho \cos\left(\theta -\psi \right)-\frac{1}{2}u\rho^{2}+{\left(\frac{R}{a}\right)}^{2}u$$

where ψ represents the perturbation function, which is expressed as a function of ρ, θ; *u* and *v* are the two “optical coordinates” of point P, that is

(13) $$u=\frac{2\pi }{\lambda }{\left(\frac{a}{R}\right)}^{2}z,\;v=\frac{2\pi }{\lambda }\left(\frac{a}{R}\right)\sqrt{x^{2}+y^{2}}$$

**Figure 4:**
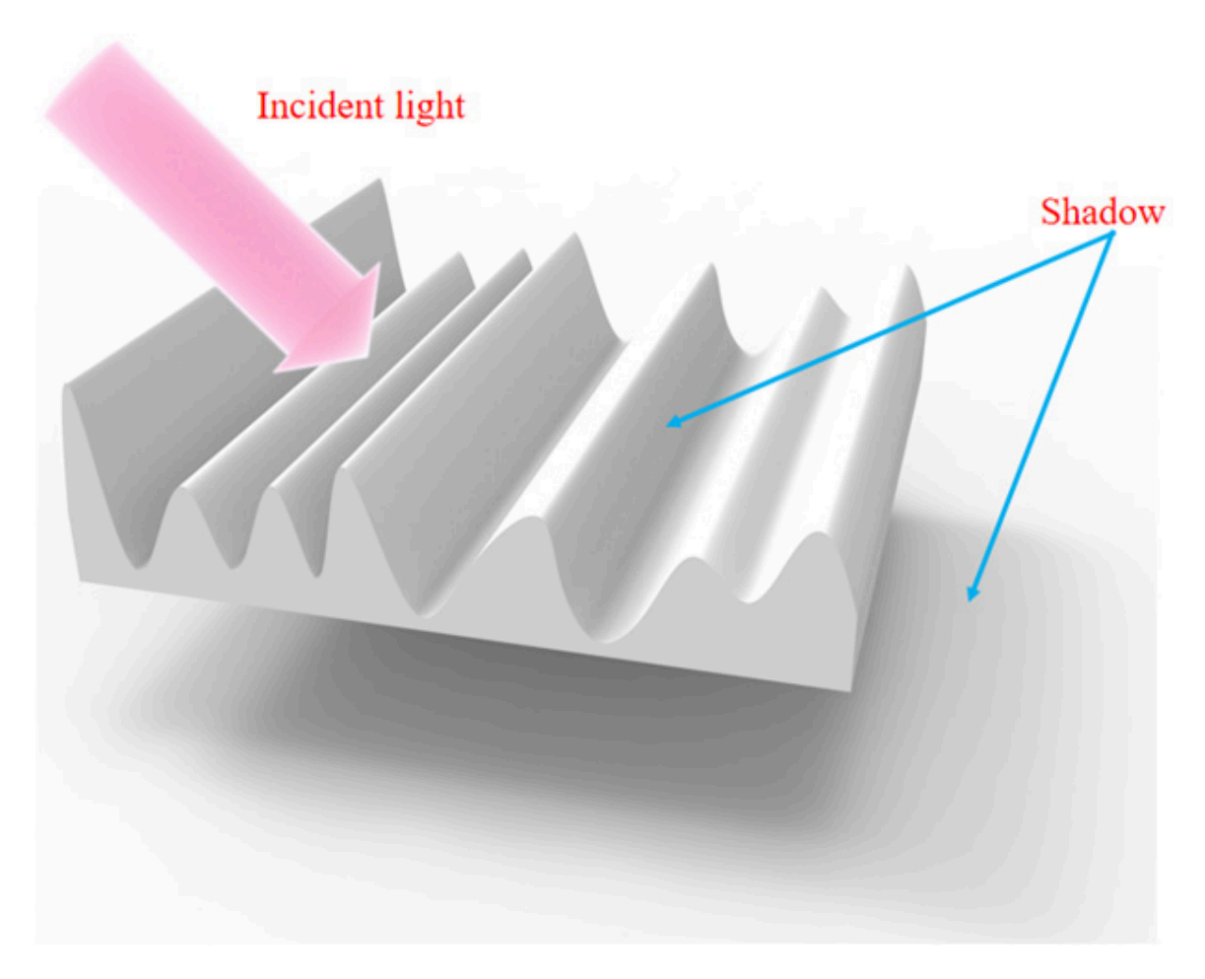
Shadowing effect at oblique incidence.

The surface element of the Gaussian reference sphere is $dS=a^{2}\rho d\rho d\theta$. According to the Huygens–Fresnel theorem, the disturbance *U*(*P*) caused by the aberration at point P (as shown in Figure 4) is obtained as

(14) $$U\left(P\right)=-\frac{1}{\lambda }\frac{Aa^{2}}{R^{2}}e^{i{\left(\frac{R}{a}\right)}^{2}u}\int_{0}^{1}\int_{0}^{2\pi }e^{i\left[kW-v\rho \cos\left(\theta -\psi \right)-\frac{1}{2}u\rho^{2}\right]}\rho d\rho d\theta$$

where *k* represents the diffraction order.

Due to the aberration caused by the spherical/aspherical surface, the light intensity of the P point is reduced by an amount *I*(*P*) of

(15) $$I\left(P\right)={\left|U\left(P\right)\right|}^{2}={\left(\frac{Aa^{2}}{\lambda R^{2}}\right)}^{2}\left|\int_{0}^{1}\int_{0}^{2\pi }e^{i\left[kW-v\rho \cos\left(\theta -\psi \right)-\frac{1}{2}u\rho^{2}\right]}\rho d\rho d\theta \right|$$

### 2.4. Influence of Shadow Effect

For general grating elements, normal incidence (incident angle is 0) is generally adopted to study their reflectivity. However, due to the special structure of spherical/aspherical mirrors, such experimental conditions are inconsistent with practical application, so we must explore the situation of oblique incidence (incident angle > 0°). In the case of oblique incidence, the surface topography produces a shadowing effect due to the linear propagation of light, as shown in Figure 4. Shadowing affects the reflectivity, which is mainly divided into two cases: when the surface morphology is similar to the wavelength of light, the polarization effect of the light needs to be considered, and due to the boundary effect of the electromagnetic field, the propagation in the grating produces wavefront distortion. Another case is the effect that the surface topography profile hinders the propagation of light. It is worth noting that for a pitch size of roughly 5 μm, observable high-order diffractions exist. The incident light is not merely absorbed due to the shadow effect, but a significant portion of energy is directed into higher diffraction orders, which fundamentally reduces the zero-order specular reflection. Taking into account the influence of shadow effect and higher-order scattering, we get the expression of actual zero-order reflection efficiency $\eta \left(d\right)$ as

(16) $$\eta \left(d\right)=\eta_{\max}-\Delta \eta \left(d\right)$$

where *d* is the period of the grating (tool mark); $\Delta \eta \left(d\right)$ is the reflectivity loss due to the shadow effect; $\eta_{\max}$ represents the reflectivity at positive incidence.

The influence of shadowing effect is modeled by rigorous coupled wave method, and the shadowing effect changes with the change in incident angle. The expression of the actual reflectivity considering the metal reflection shadowing effect is obtained as follows

(17) $$\eta \left(d\right)=\eta_{\max}\left(1-c_{m}\left(n_{r},k,\theta \right)\frac{\lambda }{d}\right)$$

where $c_{m}\left(n_{r},k,\theta \right)$ is a linear coefficient, indicating the shadow intensity; *n_r_* and *k* are constants related to the complex refractive index of metals; θ represents the incident angle; λ is the wavelength of light.

The expression of linear coefficient is

(18) $$c_{m}\left(n_{r},k,\theta \right)=c_{m}\left(n_{r},k,\theta =0\right)-a\left(n\right)\theta +b\left(n\right)\theta^{2}$$

where for aluminum, the values of *n_r_* and *k* increase with the increase in wavelength and incident angle; *a*(*n*) and *b*(*n*) depend on the complex refractive index, and are obtained by fitting the strict data of reflectivity. The values of *a*(*n*) and *b*(*n*) are shown in Table 1, which are obtained by numerical optimization.

**Figure 5:**
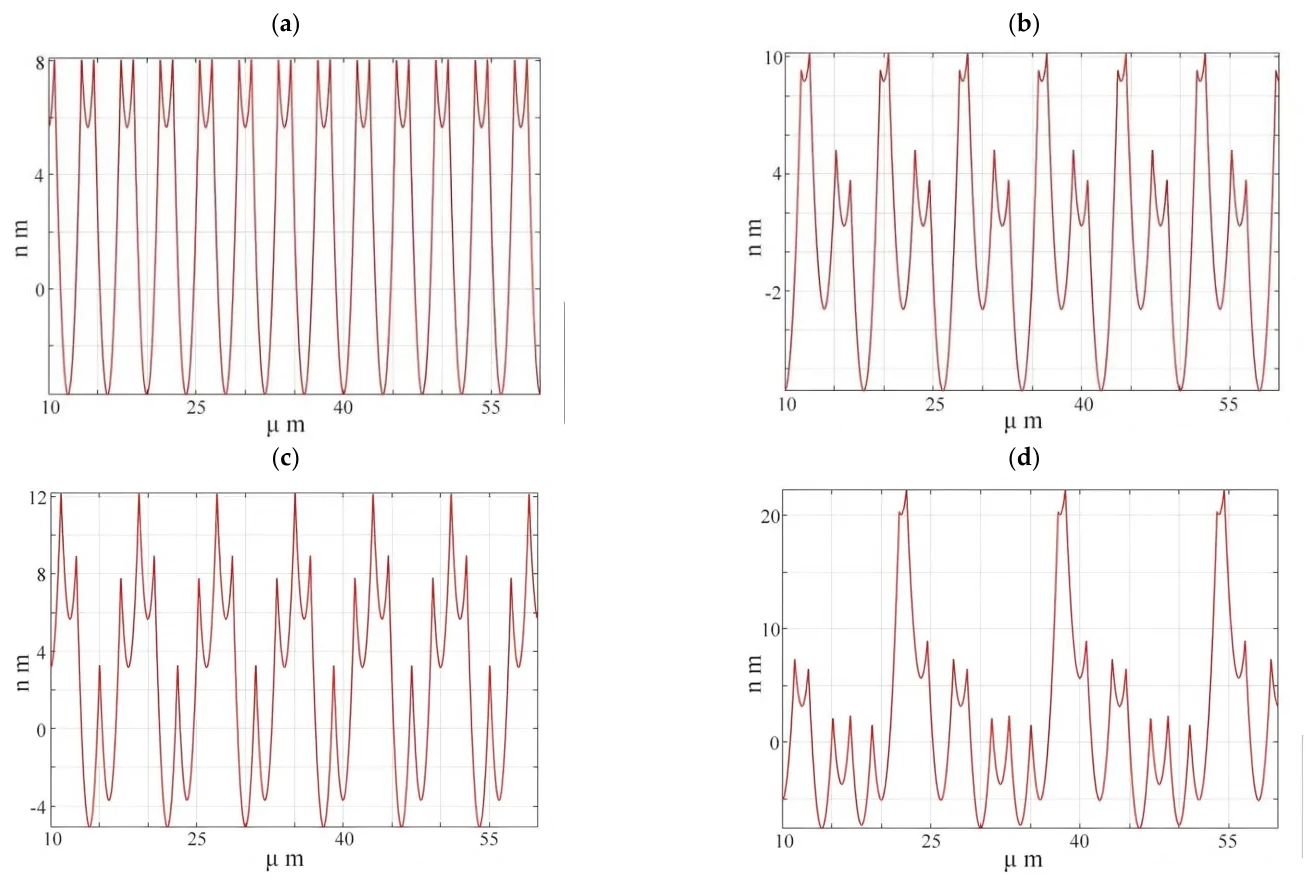
Two-dimensional cross section after superposition of different vibration modes: (**a**) Single vibration mode. (**b**) Two vibration modes. (**c**) Three vibration modes. (**d**) Four vibration modes.

**Figure 6:**
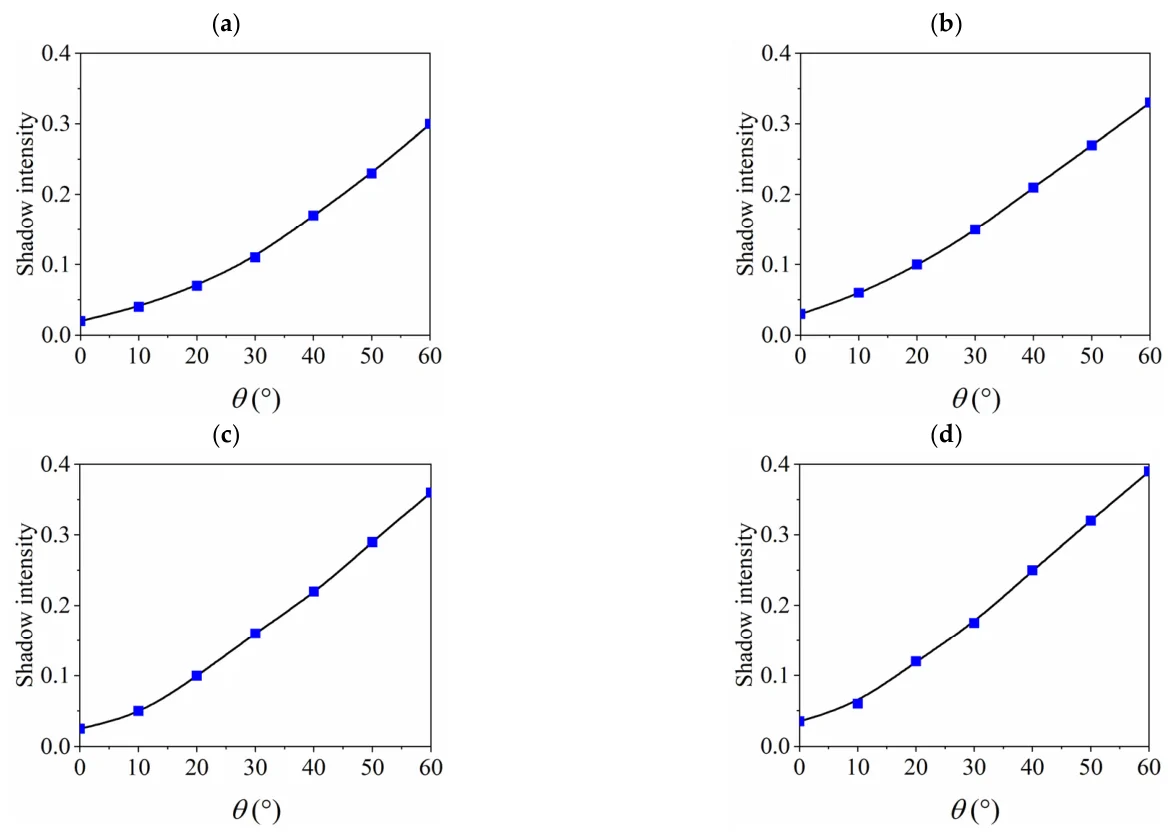
Variation trend of shadow coefficient *c_m_* corresponding to different two-dimensional cross sections: (**a**) Single vibration mode. (**b**) Two vibration modes. (**c**) Three vibration modes. (**d**) Four vibration modes.

According to our previous research work [16] on modeling the three-dimensional topography of ultra-precision turning surfaces, we can get the surface topography considering vibration, as shown in Figure 5 below, which is a schematic cross-section diagram obtained by linearly superimposing different vibration modes. According to the influence of different vibration modes on the surface topography, we can get the fitting curve of vibration surface topography and shadowing effect coefficient *c_m_*, as shown in Figure 6. It can be seen from Figure 6 that with the increase in the number of vibration modes, the surface morphology becomes more complex, and the peak-valley value of the morphology gradually increases, eventually leading to the shadow coefficient *c_m_* gradually increasing.

### 2.5. Influence of Vibration Modes on Diffraction Phenomenon and Reflectivity

#### 2.5.1. Selection of Facula Diameter

According to the above discussion, we can know that due to the particularity of spherical and aspherical shapes, the reflected trajectories of light rays in different spatial positions change, and then aberration occurs. Aberration has a non-negligible influence on reflectivity. In order to minimize this influence, the facula diameter is reasonably selected in this paper.

**Figure 7:**
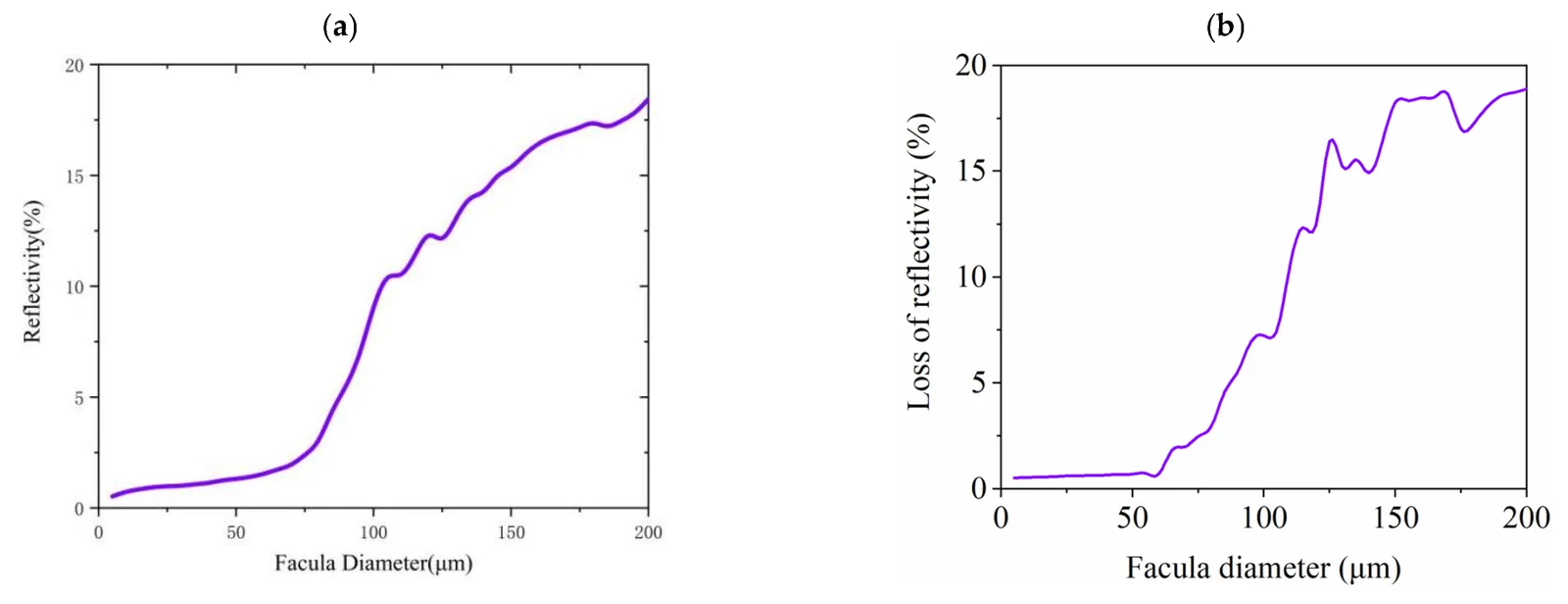
Influence of the change in facula diameter on reflectivity: (**a**) Normol incidence; (**b**) 60° incident angle.

Ensure that the incident ray wavelength is 500 nm and the surface roughness P-V value is always 15 nm. By calculations in Section 2.5, the relationship between the light facula diameter and reflectivity can be obtained/as shown in Figure 7a, for the incident angle of 0°. As can be seen from the figure, when the diameter of the light facula is less than 60 μm, the effect of aberration on the reflectivity due to the curvature of the workpiece surface is negligible. As shown in Figure 7b, the reflectivity changes with the facula diameter when the incident angle is 60°. From the figure, when the facula diameter is less than 55 μm at a large incident angle, the influence of aberration on reflectivity can be ignored. Therefore, according to the above discussion and experimental equipment, the facula diameter is set to 50 μm in this paper.

#### 2.5.2. Comprehensively Consider the Influence of Vibration Modes on Reflectivity

(1) **Influence of different numbers of vibration modes on reflectivity**

By modeling the three-dimensional surface topography, we can get the surface topography under the action of different vibration modes, as shown in Figure 5. Figure 5a–d are two-dimensional surface topography of the workpiece under the linear superposition of two vibration modes, three vibration modes, four vibration modes, and five vibration modes, respectively. From the figures, the surface P-V value increases with the increase in vibration modes. In the process of simulation, zero angle incidence is always kept, and other factors are unchanged. The simulation cutting parameters are guaranteed to be constant: the feed rate is 2 μm/r, the cutting depth is 2 μm, the spindle speed is 1200 r/min, the tool nose radius is 100 μm, and the wavelength range of incident light is set at 300 nm–1500 nm. The influence of the number of vibration modes on the turning surface reflection efficiency is obtained.

**Figure 8:**
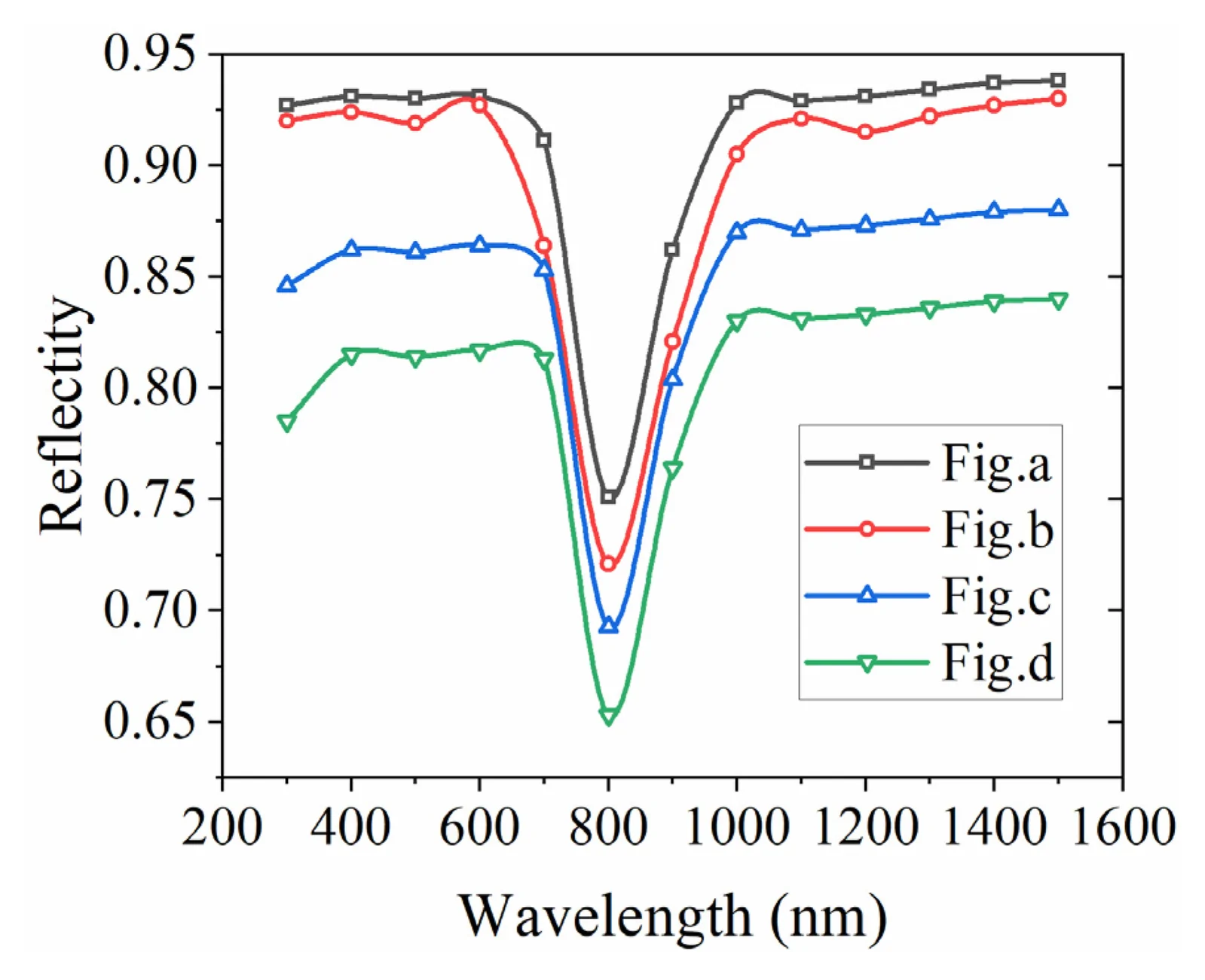
Influence of different vibration modes on surface reflectivity.

By solving the reflectivity, it can be seen from the data in Figure 8 that with the decrease in the number of vibration modes, the reflectivity in any wave band is improved. This is because with the decrease in the number of vibration modes, the peak-valley value of the surface topography decreases. According to the rigorous coupled wave method, the reflectivity model shows that with the increase in the peak-valley value, the diffraction effect intensifies and the reflectivity decreases.

(2) **Influence of different incident angles**

**Figure 9:**
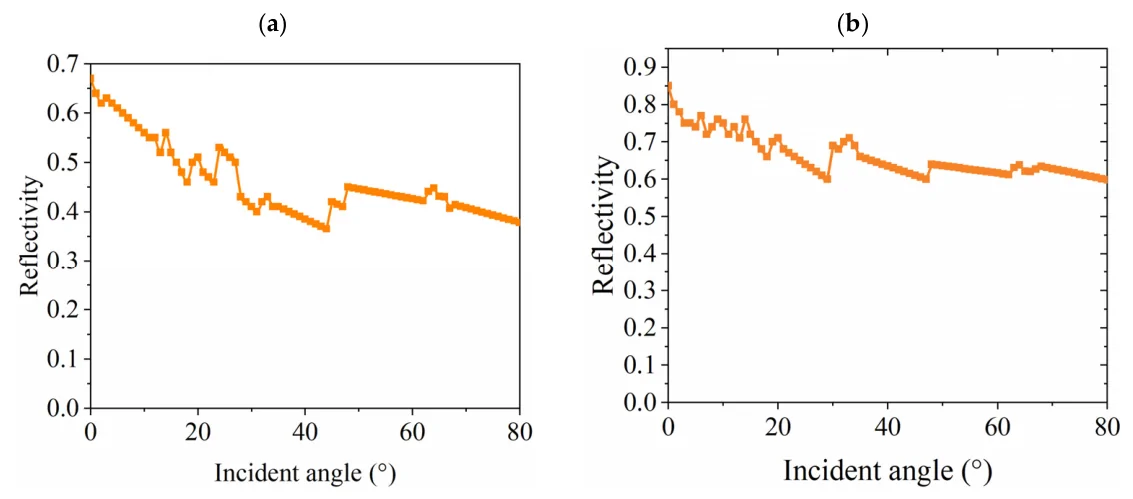
Influence of shadowing effect on reflectivity of different wavelength bands: (**a**) visible light; (**b**) near-infrared (NIR).

According to the above research on the *c_m_* coefficient, the *c_m_* coefficient increases with the increase in incident angle, which indicates that the shadowing effect increases with the increase in incident angle. As shown in Figure 9, under the condition of keeping other parameters constant, we have solved the specular light reflectivity in different wave bands. In Figure 9, the simulated cutting parameters are set as feed rate of 2 μm/r, cutting depth of 2 μm, spindle speed of 1200 r/min, tool nose radius of 100 μm, and incident light wavelengths of 500 nm, and 1200 nm, respectively. As can be seen from Figure 9a,b, whether in visible light, or near-infrared (NIR) bands, the reflectivity decreases gradually with the increase in incident angle, which is consistent with the change trend of shadowing effect intensity discussed above, indicating that the influence of shadowing effect on reflectivity cannot be ignored. This is because the smaller the wavelength, the easier it is to trap light on the surface, which intensifies the effect of shadowing effect.

**Figure 10:**
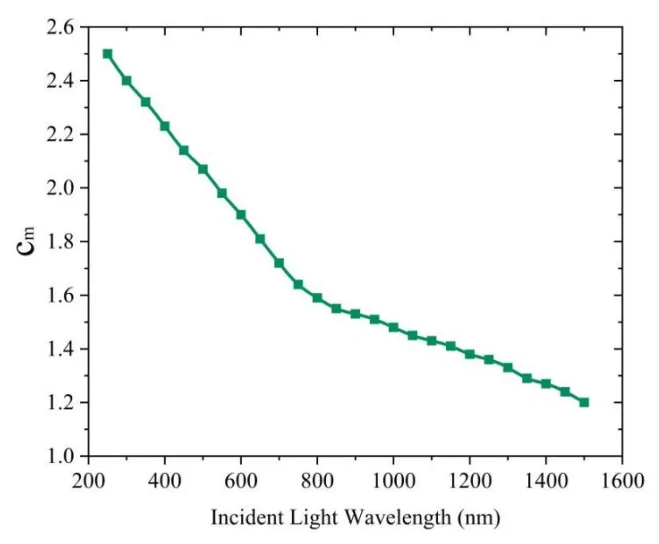
Variation in shadow coefficient with wavelength.

According to the experimental data, the change in *c_m_* value with the wavelength of incident light can be obtained by fitting the *c_m_* value, as shown in Figure 10. From the figure, it can be concluded that the *c_m_* value gradually decreases with the increase in wavelength. This result shows that the shadowing effect gradually decreases with the increase in wavelength, and this phenomenon also confirms the correctness of the simulation result in Figure 9.

(3) **Influence of different calibers of workpieces on reflectivity**

**Figure 11:**
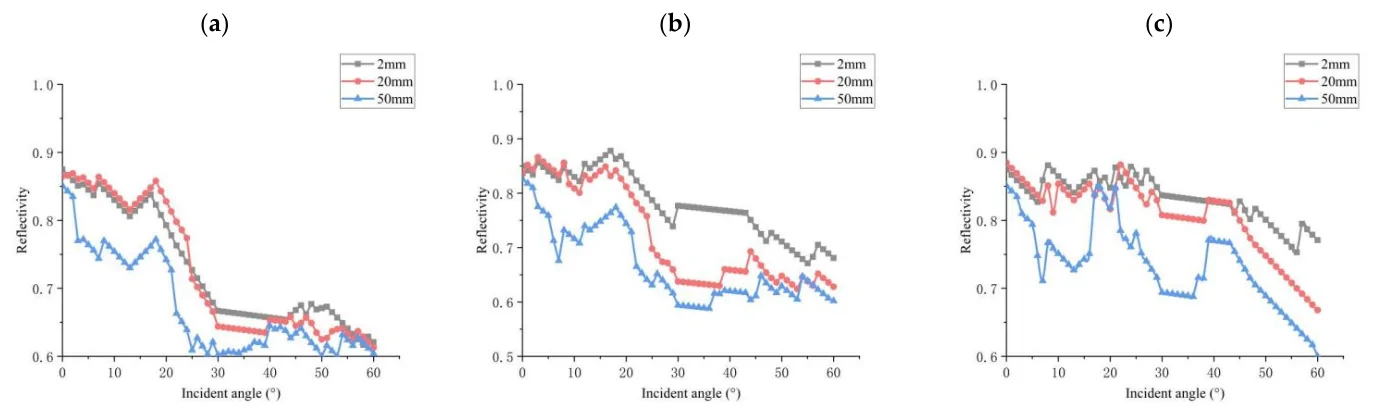
Influence of three-dimensional topography of workpiece at different positions on reflectivity: (**a**) ultraviolet (UV); (**b**) visible light; (**c**) near-infrared (NIR).

Through the author’s previous research results, we know that in the process of machining spherical/aspheric mirrors, the vibration modes between the tool and the workpiece also change with the change in the position of the tool. The law is: With the increase in caliber diameter, the cutting torque of the tool on the workpiece increases, resulting in the relative vibration of the tool–workpiece gradually increasing, and the phenomenon of bifurcation of vibration frequency (that is, a vibration frequency will be divided into two frequencies with the accumulation of energy). This law leads to the more complicated three-dimensional morphology of the machined surface, and the roughness P-V value also increases. Therefore, in this paper, the reflectivity of different caliber positions of the workpiece is calculated, and the simulation parameters in Figure 11 are set as feed rate of 2 μm/r, cutting depth of 2 μm, spindle speed of 1200 r/min, and tool nose radius of 100 μm, with other parameters unchanged. As shown in Figure 11, it can be seen that when the incident angle is 0–5°, the reflectivity changes little when the workpiece caliber size increases from 2 mm to 20 mm, but decreases obviously when it increases to 50 mm. And when the incident angle gradually increases, the reflectivity decreases more sharply with the increase in the radial dimension of the workpiece. This phenomenon shows that with the increase in the mirror caliber, the relative vibration between the tool and the workpiece intensifies, resulting in the higher surface roughness P-V. Under the condition of large incident angle, the more obvious the shadowing effect is, the greater the influence of shadowing effect on the reflectivity and the lower the reflectivity.

## 3. Adaptive Interpolation Point Planning Algorithm

### 3.1. Calculation of Cutting Vibration

#### 3.1.1. Effect of Interpolation Algorithm

Dynamic cutting forces directly affect the vibrations of the machine tool. Due to the influence of vibrations, the cutting depth and cutting width change, which in turn affects the actual cutting forces. Therefore, the dynamic cutting forces are modeled with consideration of the vibration and the variation in cutting depth and cutting width.

(1) **Influence of interpolation algorithm**

In diamond turning of aspherical surfaces, the cutting parameters dynamically change depending on the applied interpolation algorithm. The popular interpolation algorithms to plan tool paths when machining the aspherical surfaces are the equal-feed and equal-residual-height methods. This section attempts to model the effect of vibration on the cutting width and depth of cut, which are two important parameters affecting the dynamic cutting forces.

1. **Equal-feed cutting**

**Figure 12:**
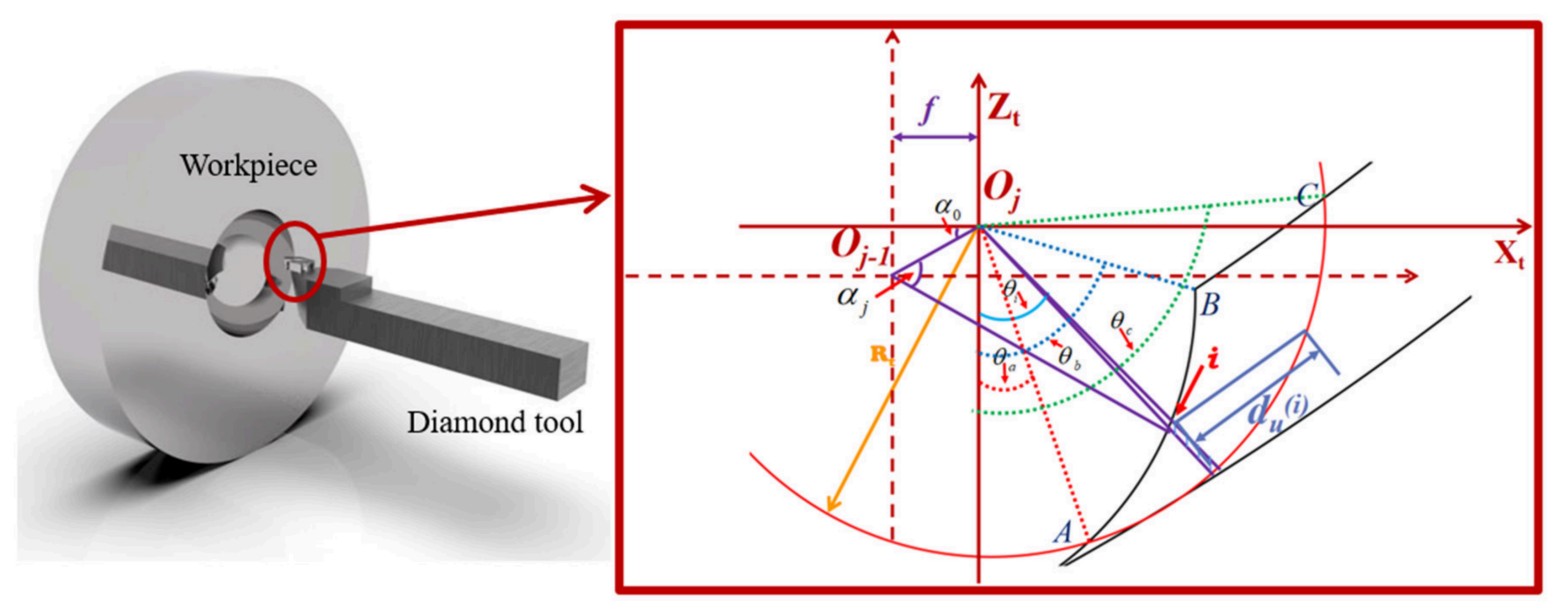
Depth of cut diagram.

The depth of cut produced by two adjacent tool paths is shown schematically in Figure 12. In this figure, *A* and *B* are the two end points of the cutting width. *O_j_* is the original point of the tool coordinate system *X_t_*–*O_j_*–*Z_t_* in response to the *j*th tool path. *O_j_*_-1_ is the original point of the tool coordinate system *X_t_*–*O_j_*_-1_–*Z_t_* with respect to the (*j*-1)th tool path. *i* is an arbitrary point on the cutting width. *f* is the feed rate. According to the geometrical relationship as described in this figure, the angle $\alpha_{i}$ is calculated as

(19) $$\alpha_{i}=\frac{\pi }{2}+\alpha_{0}-\theta_{i}-\arcsin\left[\frac{d}{R_{T}}\cdot \cos\left(\theta_{i}-\alpha_{0}\right)\right]$$

where *d* is the length of line *O_j_*_-1_*O_j_*, and $d=\sqrt{f^{2}+{\left(z_{j-1}-z_{j}\right)}^{2}}$; *z_j_*_-1_ and *z_j_* are the Z ordinates of point *O_j_*_-1_ and point *O_j_,* respectively; $\alpha_{0}$ is the included angle between the line *O_j_*_-1_*O_j_* and the X_t_-axis; $\theta_{i}$ is the included angle between the line *iO_j_* and the Z_t_-axis.

Therefore, the effective depth of cut $t_{u}^{\left(i\right)}$ at point *i* is written as

(20) $$t_{u}^{\left(i\right)}=\left\{\begin{array}{l} R_{T}-R_{T}\frac{\cos\left(\alpha_{0}-\theta_{i}-\arcsin\left[\frac{d}{R_{T}}\cdot \cos\left(\theta_{i}-\alpha_{0}\right)\right]\right)}{\cos\left(\theta_{i}-\alpha_{0}\right)}\;\;\;\theta_{i}\in \left[\theta_{a},\theta_{b}\right)\\ R_{T}-\left|\frac{z_{T}^{\left(i\right)}}{\sin\left(90^{\circ }-\theta_{i}\right)}\right|\;\;\;\theta_{i}\in \left(\theta_{b},\theta_{c}\right] \end{array}\right.$$

where $z_{T}^{\left(i\right)}$ is the Z coordinate of point *i*; $\theta_{a}$, $\theta_{b}$ and $\theta_{c}$ are the included angles between the lines *O_j_A*, *O_j_B* and *O_j_*C and the *Z_t_*-axis respectively, as marked in Figure 12; *R_T_* is the tool nose radius.

It can be seen from Equation (20) that the cutting depth grows up with the increase in *d*. Moreover, *d* increases with the enlargement of the workpiece surface slope. That is to say, the cutting depth is proportional to the surface slope of the workpiece when using the equal feed interpolation algorithm.

**Figure 13:**
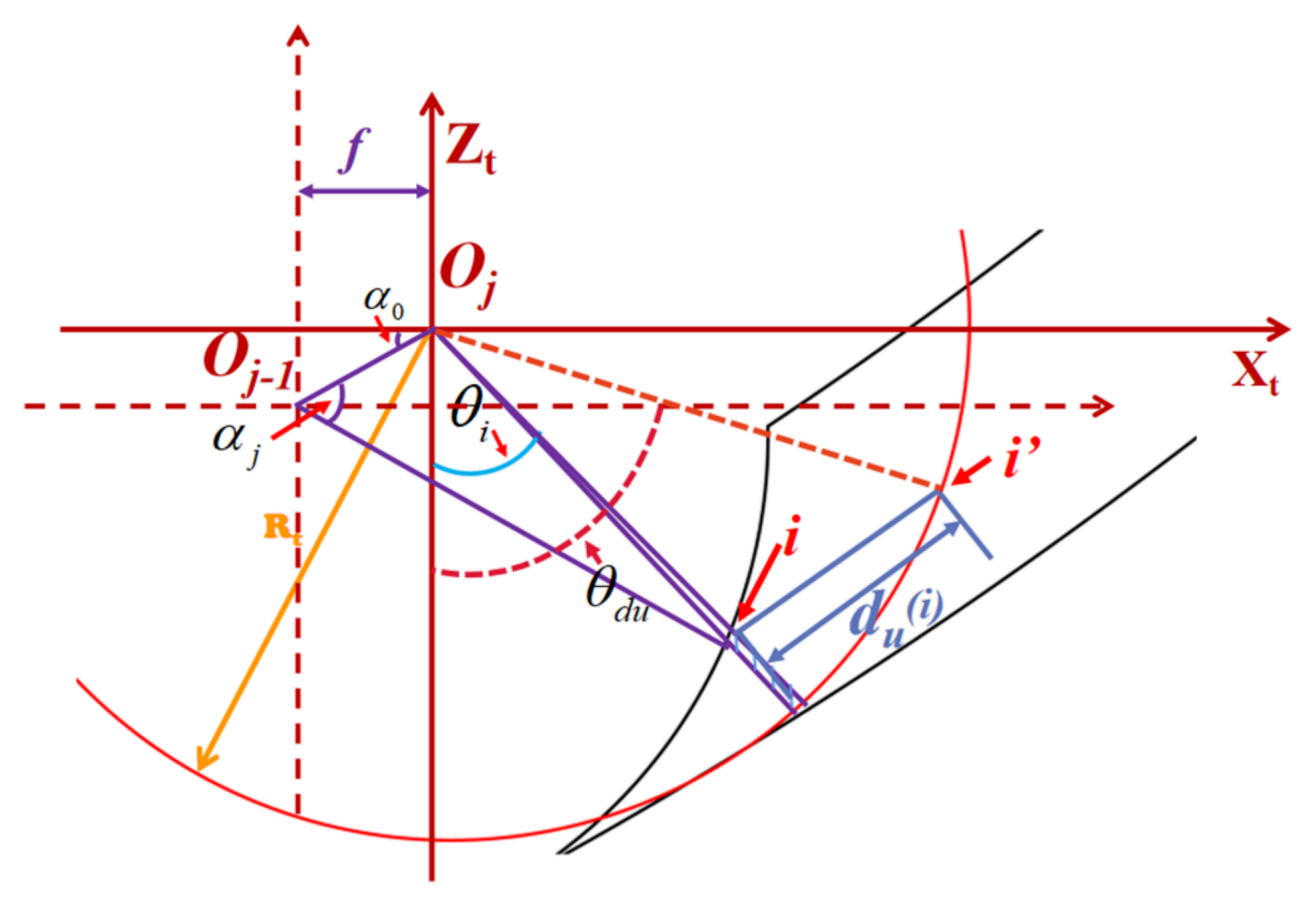
Cutting width diagram.

The expression of the effective cutting width $d_{u}^{\left(i\right)}$ at point *i* is expressed as

(21) $$d_{u}^{\left(i\right)}=\sqrt{{\left|O_{j}i\right|}^{2}+R_{T}^{2}-2\left|O_{j}i\right|\cdot R_{T}\cos\left(\theta_{du}-\theta_{i}\right)}$$

where $\theta_{du}$ is the included angle between $\left|O_{j}i^{\prime }\right|$ and the Z_t_-axis, as shown in Figure 13; *i’* is the boundary point of cutting width. $\left|O_{j}i\right|$, the distance from point *O_j_* to point *i* is

(22) $$\left|O_{j}i\right|=R_{T}\frac{\cos\left(\alpha_{0}-\theta_{i}-\arcsin\left[\frac{d}{R_{T}}\cdot \cos\left(\theta_{i}-\alpha_{0}\right)\right]\right)}{\cos\left(\theta_{i}-\alpha_{0}\right)}$$

2. **Equal-residual-height cutting**

In the equal-residual-height cutting mode, it must be ensured that the residual heights of any two adjacent cutting paths are equal, which requires the feed rate to dynamically vary with the surface slope of the workpiece. Suppose that the expression of the workpiece surface is *g*(ρ, θ).

According to the definition of equal-residual-height interpolation algorithm, the interval *L_j_* between two adjacent tool paths, *N_j_* and *N_j_*_+1_, can be expressed as

(23) $$L_{j}=\sqrt{\frac{8R_{T}\varepsilon_{t}}{1-R_{T}\zeta_{j}}}$$

where $\varepsilon_{t}$ is the residual error as required to control the residual height in planning tool paths; $\zeta_{j}$ is the surface curvature in response to the *j*-th tool path.

The $\zeta_{j}$ is formulated as

(24) $$\zeta_{j}=\min\left\{{\left.\frac{\partial^{2}g/\partial^{2}\rho }{{\left[1+\partial g/\partial \rho \right]}^{\frac{3}{2}}}\right|}_{\rho =\rho_{j}},\forall \theta \in \left[0^{\circ },360^{\circ }\right)\right\}$$

In addition, the surface slope *dz_j_* of the *j*-th tool path can be calculated as

(25) $$dz_{j}=\min\left\{{\left.\frac{\partial g}{\partial \rho }\right|}_{\rho =\rho_{j}},\forall \theta \in \left[0^{\circ },360^{\circ }\right)\right\}$$

where $\rho_{j}$ is the distance between the *j*-th tool path and the workpiece center.

The feed rate *f* in an equal residual height cutting mode can be formulated as

(26) $$f=\frac{L_{j}}{\sqrt{1+{\left(dz_{j}\right)}^{2}}}$$

By substituting Equation (26) for Equation (20), the actual depth of cut and cutting width for the equal-residual-height cutting can be determined.

(2) **Theoretical model**

According to the reported work, the tool–chip contact length *l_con_* on the rake face can be given by

(27) $$l_{con}=\frac{2\cdot h_{p}}{\tan(\vartheta )}$$

where *h_p_* is the undeformed chip thickness and its equation is

(28) $$\left\{\begin{array}{l} h_{p}=R_{T}-\sqrt{R_{T}^{2}-2f\cdot x_{t}}\;\;\;\;\;\theta_{A}\leq \theta \leq \theta_{B}\\ h_{p}=t_{\max}-\frac{t_{\max}}{f}x_{t}\;\;\;\;\;\;\;\;\;\;\;\;\;\;\;\theta_{B}\leq \theta \leq \theta_{C}\; \end{array}\right.$$

where *t_max_* is the maximum cutting depth, and ϑ is the actual shear angle; *x_t_* is the coordinates along the *X_t_* axis.

Based on the tool–chip contact length and the maximum normal stress on tool rake face, the stress distribution $\sigma \left(t_{s}\right)$ on the tool–chip contact interface can be considered as a function of the distance *t_s_* from the tool tip to the concerned point, which is formulated as

(29) $$\sigma (t_{s})=\sigma_{s2}\left[1-{\left(\frac{t_{s}}{l_{con}}\right)}^{a_{0}}\right]$$

where *a*_0_ is a power index, which is set as a constant to simplify the model; $\sigma_{s2}$ is the maximum normal stress on the rake face.

The cutting force *F_y_*_1_ perpendicular to the rake face is calculated as

(30) $$F_{y1}=\int_{0}^{l_{con}}d_{u}\sigma \left(t_{s}\right)ds$$

where *d_u_* is the cutting width.

The friction behavior at tool–chip contact interface can be divided into two parts in metal cutting, i.e., the sticking friction and sliding friction. When *t_s_* is located in the sticking friction region, the friction stress is invariable, which is approximate to the yield stress of the workpiece. When *t_s_* is located in the sliding friction region, the friction and normal stresses follow the Coulomb friction law, i.e., that the tool–chip friction coefficient *μ* is invariable.

Considering the effects of sliding friction and sticking friction, the frictional stress $\tau_{f}$ at the entire tool–chip contact interface is expressed as

(31) $$\tau_{f}=\left\{\begin{array}{l} \tau_{st},\;\;\;\;\;\;t_{s}\in \left[0,l_{d}\right]\\ \mu \cdot \sigma (t_{s}),\;t_{s}\in \left[l_{d},l_{con}\right] \end{array}\right.$$

where $\tau_{st}$ is the sticking relevant friction stress; *l_d_* is the boundary to distinguish the sliding friction and sticking friction, and ld=34lcon.

The sticking friction stress $\tau_{st}$ is written as

(32) $$\tau_{st}=\mu \cdot \sigma_{s2}(1-2^{-a_{0}})$$

Therefore, according to the friction behavior, the cutting forces *F_z_*_1_ and *F_x_*_1_ on the rake face are modeled as

(33) $$\left\{\begin{array}{l} F_{z1}=\int_{\theta_{A}}^{\theta_{C}}\left(\int_{0}^{l_{d}}\tau_{st}dl+\int_{l_{d}}^{l_{con}}\mu \cdot \sigma \left(t_{s}\right)dl\right)\cdot \cos\left(\theta_{i}\right)d\theta_{i}\\ F_{x1}=\int_{\theta_{A}}^{\theta_{C}}\left(\int_{0}^{l_{d}}\tau_{st}dl+\int_{l_{d}}^{l_{con}}\mu \cdot \sigma \left(t_{s}\right)dl\right)\cdot \sin\left(\theta_{i}\right)d\theta_{i} \end{array}\right.$$

where *F_z_*_1_ and *F_x_*_1_ are the cutting forces along the Z- and X-directions on the rake face, respectively.

In addition, the extrusion of flank face to the machined surface takes place due to the material swelling of the workpiece, which inevitably introduces the secondary elastic-plastic deformation on the machined surface. In this case, the flow stresses on the tool–workpiece contact interface can be given by

(34) $$\left\{\begin{array}{l} \tau =\tau_{s}\left(1-\frac{x}{s}\right)\sin\left(\beta_{0}\right)\\ \sigma =\frac{\tau }{\mu } \end{array}\right.$$

where *τ* and *σ* are the shear and normal stresses on the contact interface between the flank face and the machined surface, respectively; $\tau_{s}$ is the shear stress on the shear plane ahead of the active cutting edge; *s* is the material spring back; $\beta_{0}$ is the tool flank angle; *x* is the distance from the tool tip to a given point at the tool–workpiece contact interface.

According to the stress distribution predicted by Equation (34), the three-dimensional cutting forces on the flank face and cutting edge can be formulated as

(35) $$\left\{\begin{array}{l} F_{z2}=\int_{\theta_{A}}^{\theta_{C}}\int_{0}^{l_{PD}}\left(\tau \cos\left(\theta_{i}\right)-\sigma \sin\left(\theta_{i}\right)\right)R_{T}dxd\theta_{i}+\int_{\theta_{A}}^{\theta_{C}}\int_{\delta_{0}}^{\delta_{1}}\left(\tau \sin\left(\delta \right)+\sigma \cos\left(\delta \right)\right)R_{T}r_{n}d\delta d\theta_{i}\\ F_{y2}=\int_{\theta_{A}}^{\theta_{C}}\int_{0}^{l_{PD}}\left(\tau \sin\left(\theta_{i}\right)+\sigma \cos\left(\theta_{i}\right)\right)R_{T}\cos\left(\theta_{i}\right)dxd\theta_{i}+\int_{\theta_{A}}^{\theta_{C}}\int_{\delta_{0}}^{\delta_{1}}\left(-\tau \cos\left(\delta \right)+\sigma \sin\left(\delta \right)\right)R_{T}\cos\left(\theta_{i}\right)r_{n}d\delta d\theta_{i}\\ F_{x2}=\int_{\theta_{A}}^{\theta_{C}}\int_{0}^{l_{PD}}\left(\tau \sin\left(\theta_{i}\right)+\sigma \cos\left(\theta_{i}\right)\right)R_{T}\sin\left(\theta_{i}\right)dxd\theta_{i}+\int_{\theta_{A}}^{\theta_{C}}\int_{\delta_{0}}^{\delta_{1}}\left(-\tau \cos\left(\delta \right)+\sigma \sin\left(\delta \right)\right)R_{T}\sin\left(\theta_{i}\right)r_{n}d\delta d\theta_{i} \end{array}\right.$$

**Figure 14:**
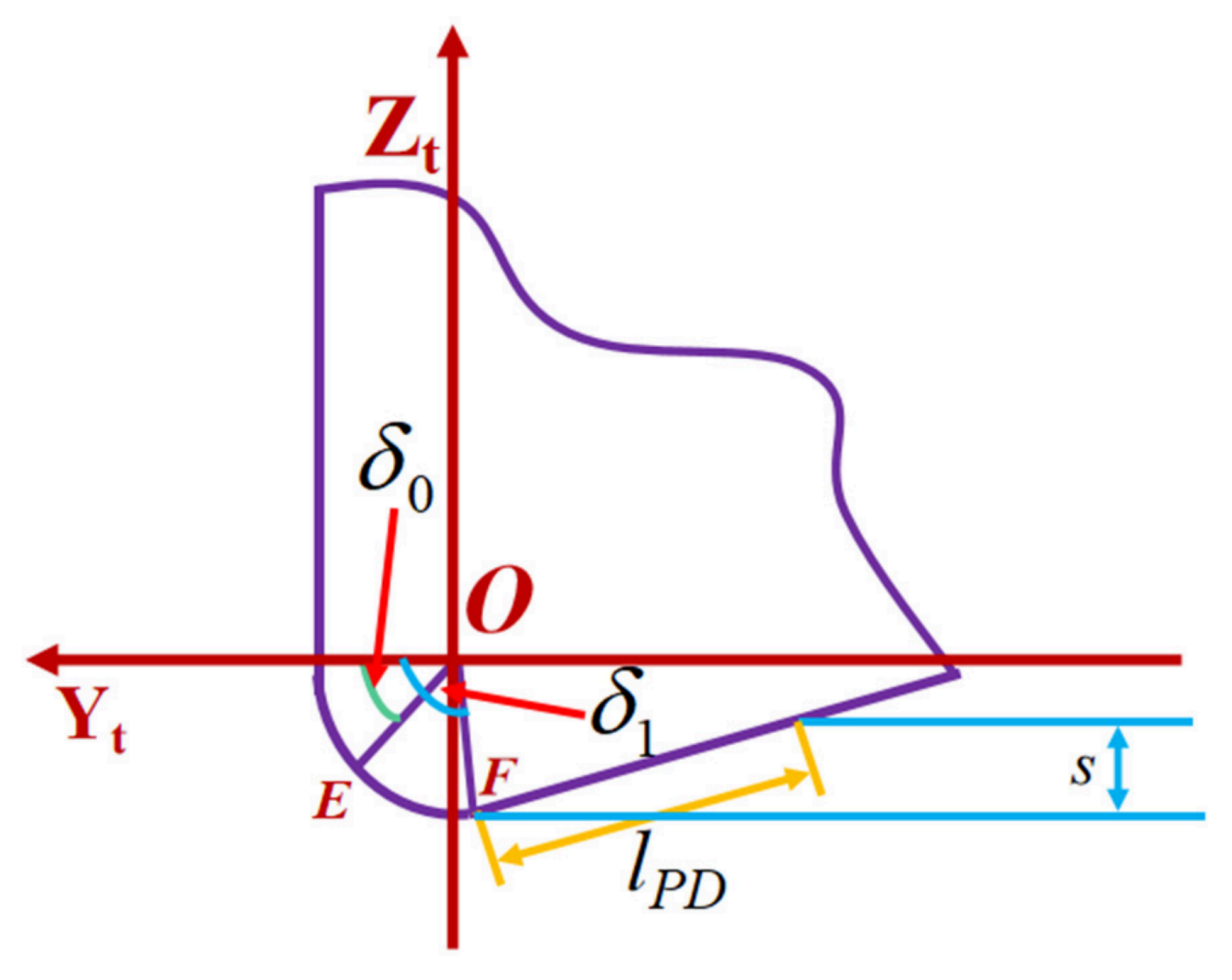
Cutting edge angle range.

The cutting edge angle range satisfies $\delta_{0}<\delta <\delta_{1}$. Here, $\delta_{0}$ is the angle between the chip separation line *OE* and the Y_t_ axis. $\delta_{1}$ is the angle between the boundary line *OF* and the Y_t_ axis, as shown in Figure 14; *l_PD_* is the total contact length between the flank face and the machined surface, and $l_{PD}=\frac{s}{\sin\left(\beta_{0}\right)}$; *F_x_*_2_, *F_y_*_2_ and *F_z_*_2_ are the three-dimensional cutting forces on the flank face; *r_n_* is the cutting edge radius of tool.

In summary, the dynamic cutting forces *F_x_*, *F_y_* and *F_z_* under no vibration and invariable cutting depth and cutting width can be expressed as

(36) $$\left\{\begin{array}{l} F_{x}=F_{x1}+F_{x2}\\ F_{y}=F_{y2}\\ F_{z}=F_{z1}+F_{z2} \end{array}\right.$$

(3) **Influence of vibration**

When the tool tip produces the translational vibration along the Z-axis, the direction of the friction force on tool rake face is related to the direction of the relative velocity Δv between the tool and the chip. In this case, the frictional stress on the rake face can be written as

(37) $$\tau_{f}=\left\{\begin{array}{l} \tau_{st},\;\;\;\;\;\;t_{s}\in \left[0,l_{d}\right]\\ \mu \cdot \sigma (t_{s})\cdot \mathrm{sgn}\left(\Delta v\right),\;\;\;\;\;\;\;\;t_{s}\in \left[l_{d},l_{con}\right] \end{array}\right.$$

In addition, once the tool–workpiece vibration takes place along the Z-direction and X-direction, the actual depth of cut *t_u_* and cutting width *d_u_* will change, and the variations $t_{u}^{\prime }$ and $d_{u}^{\prime }$ can be calculated as

(38) $$\left\{\begin{array}{l} t_{u}^{\prime }=t_{u}-Z_{t}\cdot \cos\left[\arctan\left(dz_{j}\right)\right]-Z_{w}\cdot \cos\left[\arctan\left(dz_{j}\right)\right]-X_{t}\cdot \sin\left[\arctan\left(dz_{j}\right)\right]-X_{w}\cdot \sin\left[\arctan\left(dz_{j}\right)\right]\\ d_{u}^{\prime }=d_{u}-Z_{t}\cdot \sin\left[\arctan\left(dz_{j}\right)\right]-Z_{w}\cdot \sin\left[\arctan\left(dz_{j}\right)\right]-X_{t}\cdot \cos\left[\arctan\left(dz_{j}\right)\right]-X_{w}\cdot \sin\left[\arctan\left(dz_{j}\right)\right] \end{array}\right.$$

where *v_a_* is the tool feed velocity along the *Z*-direction; *X_t_* and *X_w_* are the vibration-induced displacements of the tool and workpiece along the X-direction, respectively; *Z_t_* and *Z_w_* are the vibration-induced displacements of the tool and workpiece along the *Z*-direction, respectively. Their expressions are written as

(39) $$\left\{\begin{array}{l} X_{t}=\sum_{i=1}^{M_{1}}x_{ti}\;\;\;\;X_{w}=\sum_{i=1}^{M_{2}}x_{wi}\\ Z_{t}=\sum_{i=1}^{M_{1}}z_{ti}\;\;\;\;Z_{w}=\sum_{i=1}^{M_{2}}z_{wi} \end{array}\right.$$

where *z_ti_* is the displacement along the Z-axis of different parts in the Z-direction motion system; *z_wi_* is the displacement along the Z-axis of different parts in the X-direction motion system; *x_ti_* is the displacement along the X-axis of different parts in the Z-direction motion system; *x_wi_* is the displacement along the X-axis of different parts in the X-direction motion system; *M*_1_ is the number of components of the Z-direction motion system; *M*_2_ is the number of components of the X-direction motion system.

When the translational vibration of the machine tool parts occurs along the Y-direction, it has little influence on the cutting depth and width. Likewise, it has little influence on the cutting force, so it is not considered in this work.

When the tool tip produces the rotational vibration around the Z-axis, the X-direction coordinate changes $\Delta x_{t1}$ and $\Delta x_{w1}$ of the tool and workpiece are given by

(40) $$\left\{\begin{array}{l} \Delta x_{t1}=\sum_{i=1}^{M_{1}}\left(a_{ti}-a_{ti}\cdot \cos\left(\gamma_{ti}\right)\right)\\ \Delta x_{w1}=\sum_{i=1}^{M_{2}}\left(a_{wi}-a_{wi}\cdot \cos\left(\gamma_{wi}\right)\right) \end{array}\right.$$

where *a_ti_* is the distance from the mass center of each part of the Z-direction motion system to the tool tip along the X-axis; $\gamma_{ti}$ is the rotation angle of different parts in the Z-direction motion system around the Z-axis; *a_wi_* is the distance from the mass center of each part of the X-direction motion system to the workpiece along the X-axis; $\gamma_{wi}$ is the rotation angle of different parts in the X-direction motion system around the Z-axis.

When the tool tip produces the rotational vibration around the X-axis, the Z-direction coordinate changes $\Delta z_{t1}$ and $\Delta z_{w1}$ of the tool and workpiece are calculated as

(41) $$\left\{\begin{array}{l} \Delta z_{t1}=\sum_{i=1}^{M_{1}}\left(l_{ti}-l_{ti}\cdot \cos\left(\theta_{ti}\right)\right)\\ \Delta z_{w1}=\sum_{i=1}^{M_{2}}\left(l_{wi}-l_{wi}\cdot \cos\left(\theta_{wi}\right)\right) \end{array}\right.$$

where *l_ti_* is the distance from the mass center of each part of the Z-direction motion system to the tool tip along the Z-axis; $\theta_{ti}$ is the rotation angle of different parts in the Z-direction motion system around the X-axis; *l_wi_* is the distance from the mass center of each part of the X-direction motion system to the workpiece along the Z-axis; $\theta_{wi}$ is the rotation angle of different parts in the X-direction motion system around the X-axis.

When the tool tip produces the rotational vibration around the Y-axis, the Z-direction coordinate and X-direction coordinate changes $\left\{\begin{array}{l} \Delta x_{t2}\\ \Delta z_{t2} \end{array}\right.$ and $\left\{\begin{array}{l} \Delta x_{w2}\\ \Delta z_{w2} \end{array}\right.$ of the tool and workpiece are expressed as

(42) $$\left\{\begin{array}{l} \Delta x_{t2}=\sum_{i=1}^{M_{1}}\left(l_{ti}-l_{ti}\cdot \cos\left(\phi_{ti}\right)\right)\\ \Delta z_{t2}=\sum_{i=1}^{M_{1}}\left(l_{ti}\cdot \sin\left(\phi_{ti}\right)\right)\\ \Delta x_{w2}=\sum_{i=1}^{M_{2}}\left(l_{wi}-l_{wi}\cdot \cos\left(\phi_{wi}\right)\right)\\ \Delta z_{w2}=\sum_{i=1}^{M_{2}}\left(l_{wi}\cdot \sin\left(\phi_{wi}\right)\right) \end{array}\right.$$

where $\phi_{ti}$ is the rotation angle of different parts in the Z-direction motion system around the Y-axis; $\phi_{wi}$ is the rotation angle of different parts in the X-direction motion system around the Y-axis.

Therefore, the displacements $\left\{\begin{array}{l} X_{t}^{\prime }\\ Z_{t}^{\prime } \end{array}\right.$ and $\left\{\begin{array}{l} X_{w}^{\prime }\\ Z_{w}^{\prime } \end{array}\right.$ between the tool tip and the workpiece caused by rotational vibration are

(43) $$\left\{\begin{array}{l} X_{t}^{\prime }=\Delta x_{t1}+\Delta x_{t2}\\ Z_{t}^{\prime }=\Delta z_{t1}+\Delta z_{t2}\\ X_{w}^{\prime }=\Delta x_{w1}+\Delta x_{w2}\\ Z_{w}^{\prime }=\Delta z_{w1}+\Delta z_{w2} \end{array}\right.$$

The changes in cutting width $t_{u}^{\prime \prime }$ and cutting depth $d_{u}^{\prime \prime }$ caused by the rotational vibration are given by

(44) $$\left\{\begin{array}{l} t_{u}^{\prime \prime \prime }=t_{u}+X_{t}^{\prime }\cdot \sin\left[\arctan\left(dz_{j}\right)\right]+Z_{t}^{\prime }\cdot \cos\left[\arctan\left(dz_{j}\right)\right]+X_{w}^{\prime }\cdot \sin\left[\arctan\left(dz_{j}\right)\right]+Z_{w}^{\prime }\cdot \cos\left[\arctan\left(dz_{j}\right)\right]\\ d_{u}^{\prime \prime \prime }=d_{u}+Z_{t}^{\prime }\cdot \sin\left[\arctan\left(dz_{j}\right)\right]+X_{t}^{\prime }\cdot \cos\left[\arctan\left(dz_{j}\right)\right]+Z_{w}^{\prime }\cdot \sin\left[\arctan\left(dz_{j}\right)\right]+X_{w}^{\prime }\cdot \cos\left[\arctan\left(dz_{j}\right)\right] \end{array}\right.$$

During diamond turning operations for aspheric surface generation, the selected interpolation methodology plays a critical role in determining dynamic cutting force characteristics. The correlation between interpolation strategies and tool–workpiece vibration patterns remains unexplored in existing research. To address this knowledge gap, this study numerically modeled vibration phenomena induced by machining forces through implementation of both constant feed-rate and uniform residual height interpolation approaches. Identical cutting conditions were maintained across simulations, including a 2 μm/r feed rate, 1200 rpm spindle rotation speed, and 3 μm depth of cut. The single-crystal diamond tool employed featured a precisely ground 500 μm radius cutting tool nose.

The experimental results presented in Table 2 were acquired through application of the equal-feed interpolation method, while Table 3 documents findings from the equal-residual-height interpolation approach. Analysis of Table 2 vibration measurements reveals two distinct bifurcation phenomena occurring during the diamond tool’s radial movement from workpiece center to periphery (color-coded differentiation implemented for frequency bifurcations). Both datasets demonstrate initial bifurcation in the spindle air film’s angular stiffness vibration frequencies, as evidenced by blue-highlighted entries. Subsequent bifurcation manifests in the X-axis guideway oil film’s angular stiffness vibration frequencies, indicated through green-coded data points. The equal-residual-height method produces gradual variations in the depth of cut, consequently maintaining relatively stable dynamic cutting forces throughout the process. This operational characteristic results in frequency bifurcation manifests at machining locations positioned beyond 25 mm from the workpiece centerline, as indicated by the red-highlighted positional data in Table 3. Nevertheless, the implemented algorithm demonstrates substantial vibration suppression capabilities, as evidenced by the amplitude measurements documented in Table 3. These findings substantiate the critical requirement for developing optimized interpolation algorithms to effectively mitigate machining vibrations.

In this table, *r_n_* denotes the distance from the cutting position to the center on the workpiece surface.

#### 3.1.2. Investigation of Vibration Signal Emergence Patterns Through Stochastic Simulation Techniques

Vibration frequency bifurcation occurs when system parameters or external forces alter, leading to abrupt shifts in vibration frequencies. This instability typically appears as rapid transitions in frequency quantities during dynamic system operations. Detailed numerical results illustrating this phenomenon can be found in Table 2 and Table 3, where color-coded data highlights critical variations. As previously analyzed, machining parameter adjustments induce distinct vibration bifurcation behaviors at various surface locations of the workpiece. This observation necessitates comprehensive investigation into how vibrational characteristics correlate with spatial positioning during material removal processes. To achieve this analytical goal, a stochastic big data simulation approach was implemented with the following parameter ranges: depth of cut (2–10 μm), rotational velocity (400–2000 r/min), feed rate (0.5–5 μm/revolution), and radial distance from workpiece center (0–100 mm).

**Figure 15:**
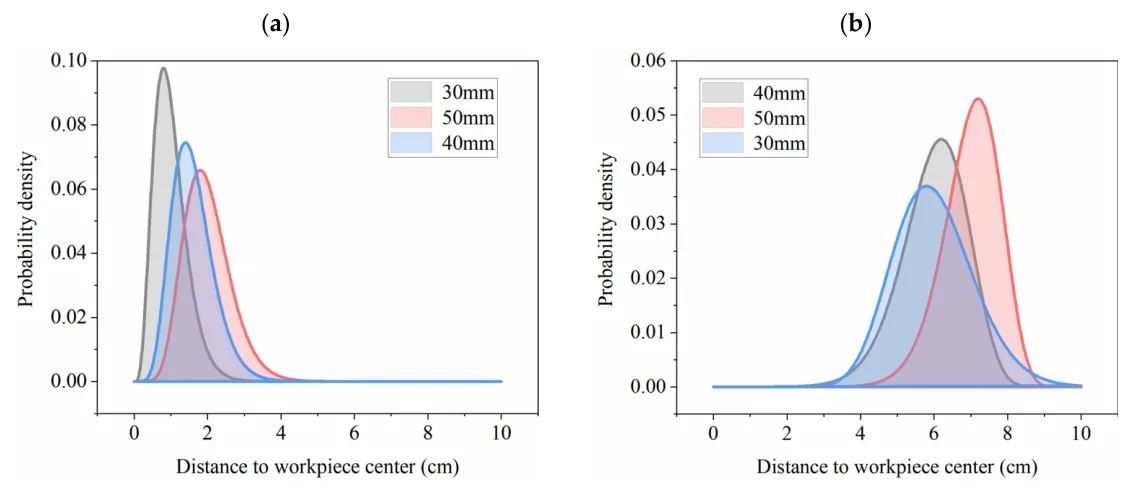
Influence of spherical curvature radius on vibrational frequency bifurcation probability density: (**a**) Initial bifurcation emergence. (**b**) Secondary bifurcation development.

Numerical simulations of spherical surface machining processes revealed distinct statistical distribution characteristics of vibration frequency bifurcation across varying spherical radii under consistent sagittal height conditions, as illustrated in Figure 15. As evidenced by the trend lines in this graphical representation, reduced spherical radii correlate with earlier bifurcation occurrences in vibration frequencies at machining locations proximal to the geometric center. This phenomenon arises from the progressive elevation of surface inclination angles and incremental expansion of the machined region accompanying reductions in workpiece curvature dimensions. These geometric modifications induce substantial torsional loads on both the workpiece and spindle system during near-central machining operations, precipitating premature manifestation of vibrational frequency bifurcation events.

**Figure 16:**
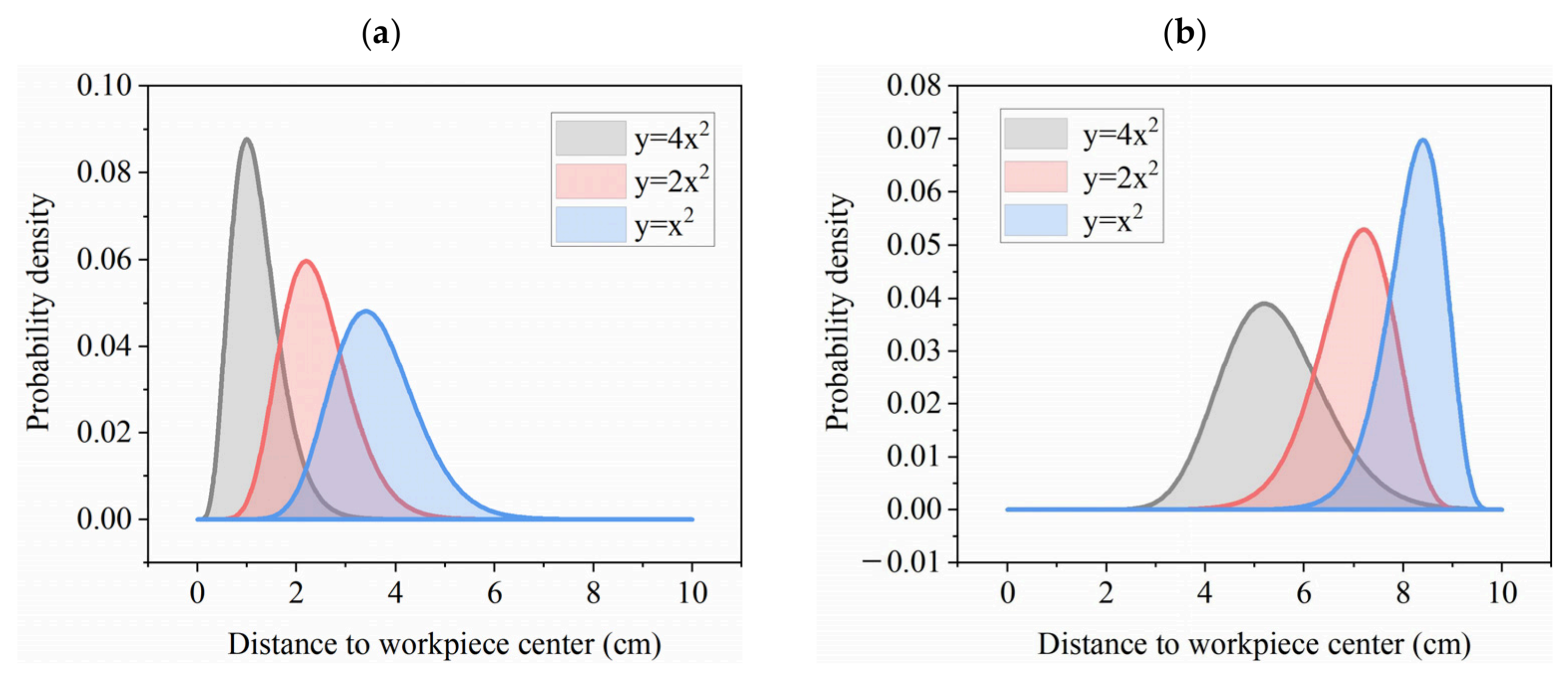
Impact of parabolic generatrix configuration on vibrational frequency bifurcation distribution: (**a**) Primary bifurcation manifestation at cutting location. (**b**) Subsequent bifurcation formation at cutting interface.

When modeling the machining of aspherical surfaces such as parabolic profiles, the sagittal height is presumed equivalent to that utilized in spherical surface processing. Notably, the gradient of parabolic contours exhibits substantial variation across distinct meridian equations, thereby altering the torque exerted on the spindle. Therefore, stochastic big data modeling that accounts for varying meridian equations becomes essential for forecasting the probability distribution of vibration frequency bifurcation. As illustrated in Figure 16, the parabolic formulations are standardized into a uniform expression: $y=a\cdot x^{2}$ (a > 0). As parameter a increases, the occurrence positions of primary and secondary vibration bifurcations shift closer to the workpiece’s central axis. The established vibration model in Section 2.1 reveals that elevated a values correspond to steeper parabolic surface gradients under identical machining conditions, inducing amplified torsional forces on both the spindle assembly and cutting tool. This mechanical relationship explains why both bifurcation phenomena manifest nearer to the workpiece’s central region when operating with higher a values.

This investigation employed sensitivity gradient analysis and sensitivity factor quantification to evaluate parameter influences on vibration bifurcations. The analyzed variables included cutting depth, feed rate, rotational velocity, tool nose radius, and surface inclination angle.

The computational outcomes are compiled in Table 4.

As indicated by the information presented in Table 4, the relative significance of operational parameters affecting vibration frequency bifurcation follows this decreasing priority sequence: cutting depth, surface inclination of workpiece, rotational velocity of spindle, radius of cutting tool tip, and feed rate. Consequently, careful selection of an appropriate interpolation algorithm becomes essential, particularly when accounting for variations in the workpiece surface slope.

### 3.2. Solution of the Limit Cutting Depth

In this paper, we plan the position of interpolation point by studying the limit cutting depth that causes the vibration bifurcation phenomenon, and generate a continuous tool path according to the discrete tool position.

There are two traditional interpolation point planning methods, namely, equal feed interpolation algorithm and equal residual height interpolation algorithm. According to the author’s previous research results, we can get the change in vibration frequency under two interpolation algorithms. As shown in Table 2 and Table 3, it can be seen that by using the equal residual height interpolation algorithm, the vibration frequency bifurcation phenomenon (the blue and green parts in the table, the number of vibration frequencies bifurcates from one to two, which is called the vibration bifurcation phenomenon in this paper) occurs when the workpiece diameter is 25 mm, while the equal feed interpolation occurs when the workpiece diameter is 15 mm, which indicates that the equal residual height interpolation algorithm has a good suppression effect on the vibration of the system. This phenomenon further shows that the proper interpolation algorithm can obviously restrain the bifurcation of vibration frequency. However, even if the interpolation algorithm with equal residual height is adopted, the vibration frequency will still diverge, which will worsen the surface morphology of the workpiece. In order to suppress the bifurcation phenomenon of vibration, this paper explores a new interpolation point planning method.

**Figure 17:**
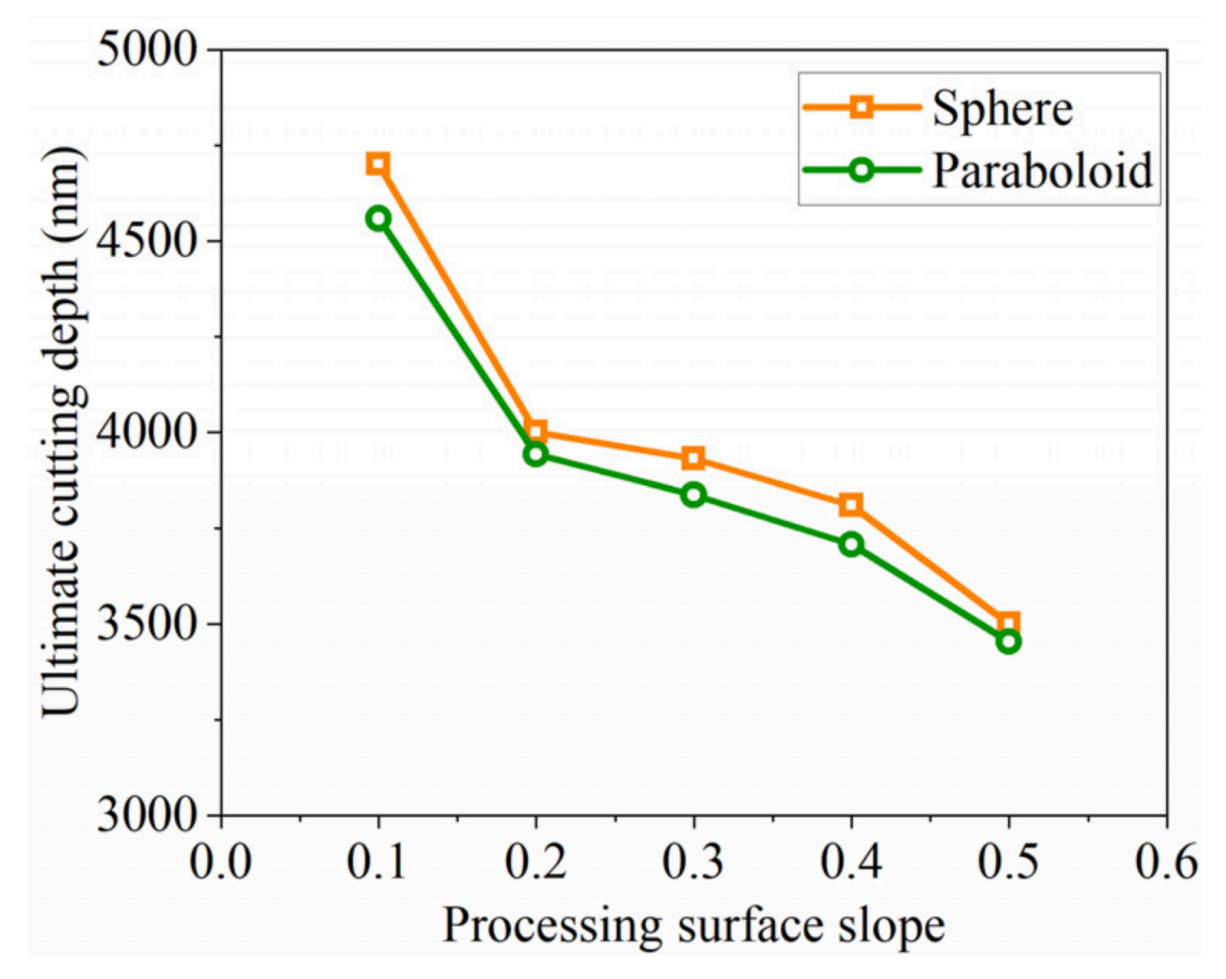
The variation in cutting depth with the slope of workpiece.

According to the author’s previous research results, the bifurcation phenomenon of vibration frequency is related to dynamic cutting force, which is directly affected by cutting depth, so that the limit cutting depth that causes the bifurcation of vibration frequency can be obtained. As shown in Figure 17, which studies the limit cutting depth of spherical surface and paraboloid of revolution respectively, with the increase in the slope of the machined workpiece surface, the limit depth of vibration bifurcation phenomenon gradually decreases, and the traditional interpolation algorithm cannot change the cutting depth with the slope of the workpiece. In this case, when the actual cutting depth exceeds the limit cutting depth, the vibration frequency bifurcation phenomenon occurs in the system. Therefore, the traditional interpolation algorithm of equal feed and equal residual height cannot change the cutting depth according to the slope of the machined surface, which indicates that it is necessary to propose a new interpolation point planning algorithm.

### 3.3. Determination of Adaptive Interpolation Point Planning Algorithm

**Figure 18:**
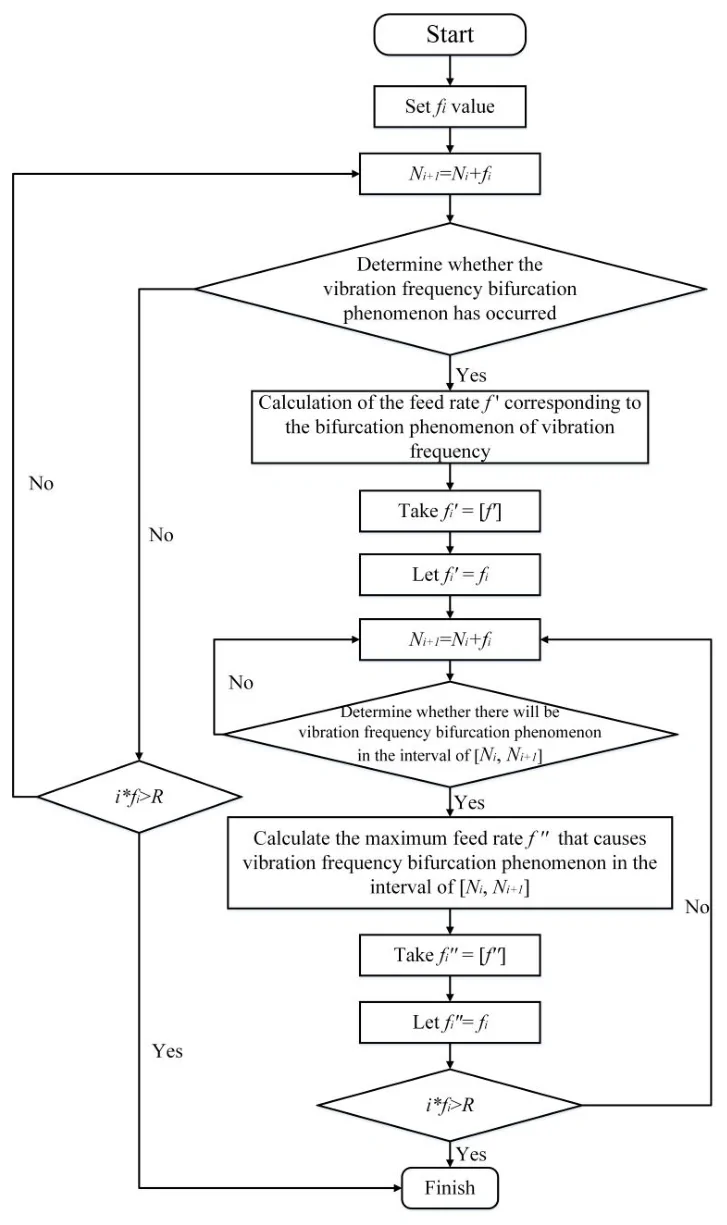
Flow chart of interpolation point position optimization (The “*” in the figure is a multiplication sign).

In order to limit the influence of system vibration on optical surface reflectivity, this paper uses local optimization algorithm to plan the position of each interpolation point according to the limit cutting depth of the bifurcation of vibration frequency obtained above. The flow chart of optimization algorithm for interpolation point position on meridian is shown in Figure 18. As the computational complexity increases with the increase in the total interpolation points in meridian, a feed rate can be set first, and the feed rate can be used to plan the position of interpolation points, so as to reduce the computational load. According to the quantitative relationship between feed rate and reflectivity explored by He [16,17], the value of the set feed rate can be determined according to the ideal reflectivity. The meridian is divided into several areas by the set feed rate. When the set feed rate cannot meet the requirements of vibration suppression, the limit cutting depth at the specified slope of the workpiece surface is calculated according to the spherical/aspheric mirror parameters provided by the customer. Because there is a corresponding relationship between the cutting depth and the feed rate, the limit feed rate corresponding to the limit cutting depth is calculated, and the new feed rate is used for new area division. By analogy, the optimization algorithm continuously calculates a series of interpolation points, divides the meridian of the workpiece into several interpolation points with the optimal feed rate, and, finally, connects each interpolation point with a linear function.

#### 3.3.1. Specific Steps of the Algorithm

According to the author’s previous research results, it can be found that when machining spherical/aspheric surfaces, the bifurcation of vibration frequency hardly occurs when the radial dimension is small; so, in order to improve the machining efficiency, the set feed rate can be used for machining at the smaller radial dimension. When the machining surface diameter gradually increases, the phenomenon of vibration bifurcation will occur. At this time, it is necessary to calculate the limit cutting depth in different intervals, and then plan the corresponding feed rate and determine the position of interpolation points. The specific steps are as follows:

(1) Firstly, according to the requirement of ideal reflectivity on surface roughness, set the initial feed rate value *f_i_*, divide the regions along the meridian with the initial feed rate value, and calculate whether the vibration frequency bifurcation phenomenon occurs in each region in sequence.
(2) If the vibration frequency bifurcation phenomenon does not occur (the number of general solutions for non-homogeneous differential equations remains unchanged), continue to plan the interpolation points according to the initial feed rate value.

(45) $$\left\{\begin{array}{l} M_{n}v_{i,tt}+C_{n}v_{i,t}+K_{n}v_{i}=f_{i}\\ J_{n}v_{\theta i,tt}+C_{\theta n}v_{\theta i,t}+K_{\theta n}v_{\theta i}=T_{qi} \end{array}\right.$$

(46) $$\left\{\begin{array}{l} m_{1}\ddot{z}+\left(c_{gz}+C_{gz}\right)\dot{z}+\left(k_{gz}+K_{gz}\right)z=F_{oz}+F_{encoder}\\ m_{1}\ddot{y}+\left(c_{gy}+C_{gy}\right)\dot{y}+\left(k_{gy}+K_{ay}\right)y=G_{holder}+F_{oy}\\ m_{1}\ddot{x}+\left(c_{gx}+C_{gx}\right)\dot{x}+\left(k_{gx}+K_{gx}\right)x=F_{ox} \end{array}\right.$$

(3) If the vibration frequency bifurcation occurs (as the non-homogeneous term (i.e., external force term) increases, the number of general solutions to non-homogeneous differential equations also increases), calculate the maximum cutting depth and corresponding feed rate value within the interval according to the following formula, round the feed rate value to the new feed rate, and divide the remaining area by the feed rate value again.

(47) $$t_{u}^{\left(i\right)}=\left\{\begin{array}{l} R_{T}-R_{T}\frac{\cos\left(\alpha_{0}-\theta_{i}-\arcsin\left[\frac{d}{R_{T}}\cdot \cos\left(\theta_{i}-\alpha_{0}\right)\right]\right)}{\cos\left(\theta_{i}-\alpha_{0}\right)}\;\;\;\;\theta_{i}\in \left[\theta_{a},\theta_{b}\right)\\ R_{T}-\left|\frac{z_{T}^{\left(i\right)}}{\sin\left(90^{\circ }-\theta_{i}\right)}\right|\;\;\;\;\theta_{i}\in \left(\theta_{b},\theta_{c}\right] \end{array}\right.$$

(48) $$d_{u}^{\left(i\right)}=\sqrt{{\left|O_{j}i\right|}^{2}+R_{T}^{2}-2\left|O_{j}i\right|\cdot R_{T}\cos\left(\theta_{du}-\theta_{i}\right)}$$

(4) In the next area, continue to calculate the limit cutting depth and feed rate, and continue to divide the remaining areas with this feed rate.

Repeat step (4) until the following condition (3) is satisfied

(49) $$s.t.\;\sum_{k=1}^{M_{i}}f_{i}\left(t\right)\geq R$$

This condition indicates that the sum of feed rate amounts is greater than or equal to the mirror caliber *R*, and *M_i_* is the number of interpolation points.

#### 3.3.2. Selection of Initial Feed Rate Value

According to the above interpolation point planning algorithm, we should first choose the appropriate feed rate *f_i_*. The selection of the initial feed rate should be determined in combination with the requirement of the reflectivity of the machined surface and the actual machining time. In order to improve the machining efficiency and realize efficient machining, it is necessary to ensure that the surface reflectivity reaches the theoretical value. The processing time *t* can be determined by the following formula

(50) $$t=\frac{R}{\sum_{i=1}^{n/60}f_{i}}$$

where *R* is the caliber of the workpiece; *n* represents the rotational speed.

Constraint condition is

(51) $$s.t.\left\{\begin{array}{l} \mathrm{Priority}\;\mathrm{guarantee}\;\max\left(r_{reflective}\right)\\ \min\left(t\right) \end{array}\right.$$

In the formula, *r_reflective_* represents the highest reflectivity corresponding to specific tool parameters and cutting parameters, which can be obtained by modeling according to the formation law of machined surface topography and strict coupled wave method. Priority guarantee means to guarantee this condition first. After modeling the three-dimensional topography of the surface and calculating the reflectivity, we obtained that when the nose radius of the tool is 100 μm and the feed rate is selected by 0.54 μm/r, the surface roughness reaches a minimum value and the reflectivity reaches an ideal value (e.g., approximately 93.6% for bare aluminum at a 500 nm wavelength). This ideal reflectivity is calculated based on the Fresnel equations, representing the theoretical limit of specular reflection for the pure material, which is essentially 100% minus the intrinsic light absorption rate of the aluminum substrate. Therefore, the initial feed rate is set to 0.5 μm/r.

### 3.4. Simulation of Surface Reflectivity of Workpiece

**Figure 19:**
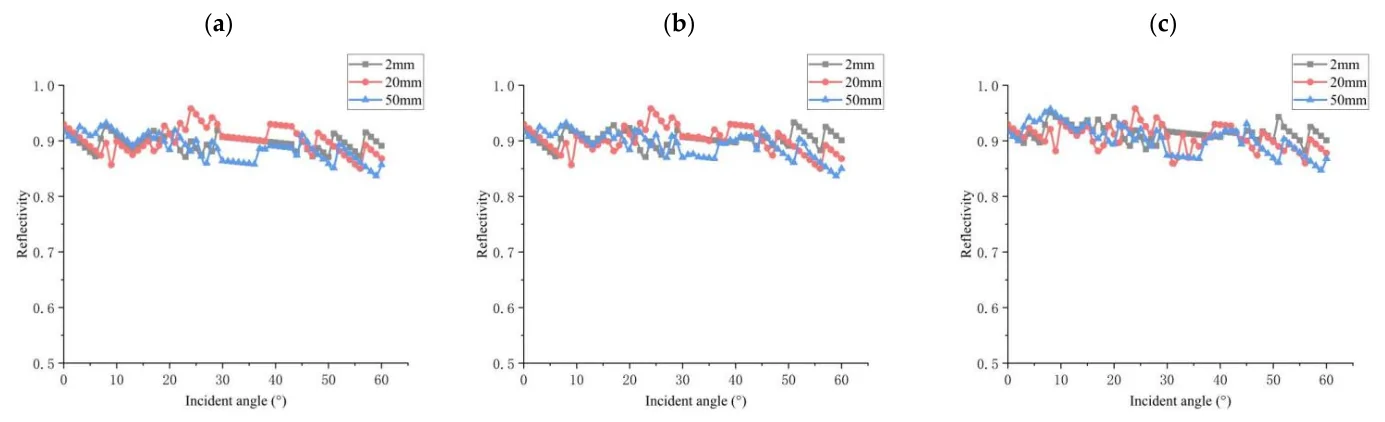
Influence of three-dimensional topography of workpiece at different positions on reflectivity: (**a**) ultraviolet (UV); (**b**) visible light; (**c**) near-infrared (NIR).

Through the interpolation algorithm in the previous section, we can get the distribution of interpolation points on the surface of the workpiece. According to the interpolation point planning, we can get the vibration at different positions of the workpiece. The vibration directly affects the three-dimensional topography of the mirror surface, and then according to the previous research results [17], we can get the three-dimensional topography of the surface. Based on the three-dimensional surface topography modeling method and the reflectivity models of S-wave and P-wave in Section 1, we can get the trend curve of surface reflectivity with wavelength, as shown in Figure 19. In Figure 19, the simulation parameters are set as feed rate of 0.5 μm/r, cutting depth of 2 μm, spindle speed of 1200 r/min, tool nose radius of 100 μm, and incident light wavelengths of 350 nm, 500 nm and 1200 nm.

From the curve in Figure 19, it can be seen that with the increase in incident angle, the reflectivity always keeps above 0.9. Moreover, even when machining large-caliber workpieces, the surface reflectivity can be kept above 0.9, which is a satisfactory result, indicating that the vibration is weakened and the contribution to the three-dimensional surface topography is reduced. As can be seen from the figure, the maximum reflectivity can reach the ideal reflectivity (that is, 100% minus the absorption rate of the material to the incident light). On the other hand, the decrease in roughness also reduces the influence of shadowing effect on reflectivity. As can be seen from Figure 19, with the gradual increase in incident angle, the reflectivity in any wavelength band does not change obviously, which shows that the improvement of surface quality makes shadowing effect well suppressed.

As shown in Figure 19, a reasonable interpolation programming algorithm can effectively improve the reflectivity of the mirror. We can find the reason for this phenomenon by analyzing the vibration modes between the tool and the workpiece. The simulation parameters in Table 5 are feed rate of 0.5 μm/r, cutting depth of 2 μm, spindle speed of 1200 r/min and tool nose radius of 100 μm. As the interpolation point planning algorithm is adopted, the influence of the workpiece’s caliber on the vibration of the machine tool system is reduced. As shown in Table 5, it can be seen that the amplitude of vibration can always be kept at a stable value with the change in caliber size. It can be seen from Table 5 that using the new interpolation point planning algorithm, the radial dimension of the workpiece has little influence on the reflectivity, so that any point on the spherical/aspherical mirror can reach the required reflectivity.

## 4. Experiment

### 4.1. Reflectivity Measuring Instrument

The measurement of surface reflection efficiency is an indirect measurement, which cannot be affected by other stray light. This puts forward two requirements for the measurement system. First, the measurement system needs to have a suitable light path structure to ensure that the detector can collect all the light flux to be measured. Second, the measurement system needs to be sealed to shield the influence of other stray light.

To sum up, this paper uses Olympus USPM-RU-W near-infrared spectrometer (Olympus Corporation, Tokyo, Japan) to measure the reflectivity of concave mirror. Through the above theoretical calculation, the diameter of the light facula is selected as 50 μm. In order to verify the effect of different incident angles on the reflectivity, the experimental incident ray angles are selected from 0° to 80°. The light wave band is selected as: 300 nm–1500 nm.

To validate the effectiveness of the proposed adaptive interpolation point planning algorithm in reducing vibration, a surface roughness measurement instrument (Zygo white light interferometer, AMETEK Group, Berwyn, PA, USA) was utilized directly on the machined substrates. The reduced surface roughness (Ra/PV) directly correlates to the suppression of system vibrations. Furthermore, to measure the specular reflectivity of the curved (spherical and parabolic) reflectors and compare it with the RCWA flat-surface assumption simulations, a customized reflectivity measurement setup was established. The system configuration comprises an Olympus USPM-RU-W near-infrared spectrometer and a curvature-compensating fixture designed for curved surfaces. This setup isolates the zero-order specular reflection for accurate evaluation.

### 4.2. Establishment of Cutting Experiment

The ultra-precision lathe used in this paper is a four-axis ultra-precision lathe developed by our school’s Institute of Precision. Taking into account the common cutting parameters of the ultra-precision lathe used, the feed rate is 0.5 µm/r, the cutting depth is 2 µm, and the spindle speed is 1200 r/min.

The material of the workpiece used in this experiment is RSA6061 material, which is formed by powder metallurgy rapid solidification method, with an average grain of 2–3 μm, which avoids the influence of large grain 6061 aluminum alloy on system vibration due to grain boundaries and hard points.

## 5. Validation of Interpolation Algorithm Effectiveness

### 5.1. Verification of the Effectiveness of Reducing Cutting Vibration

**Figure 20:**
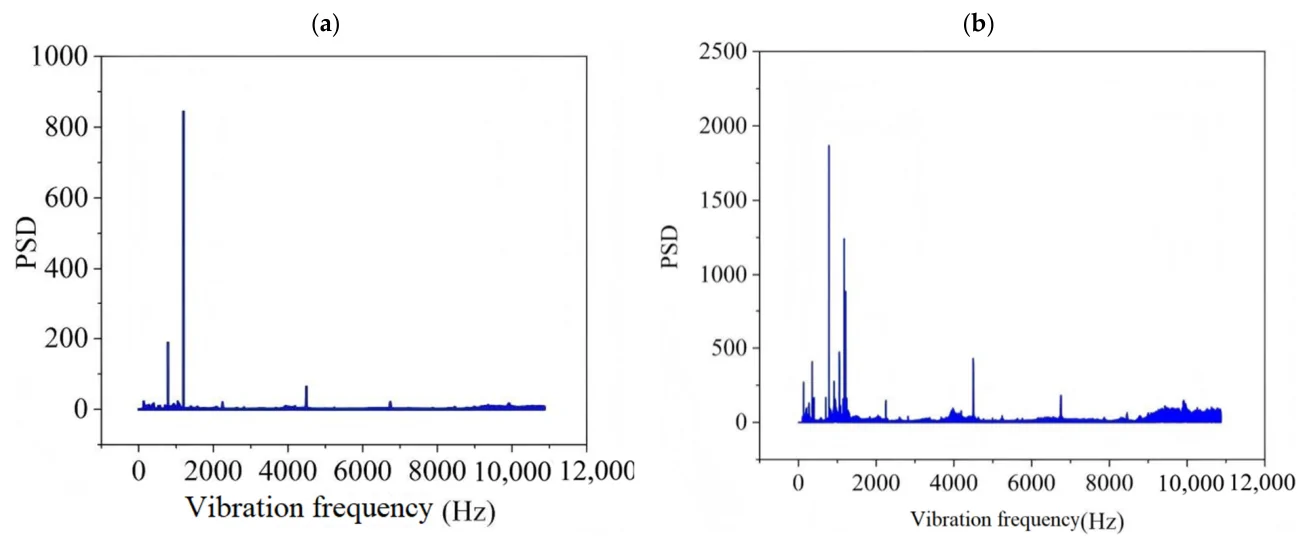
Comparison of vibration modes between adaptive interpolation algorithm and equal residual height interpolation algorithm in machining process: (**a**) Adaptive interpolation algorithm. (**b**) Equal residual height interpolation algorithm.

In order to verify the effectiveness of the adaptive interpolation algorithm, this section measures the tool–workpiece vibration mode in the machining process using the new interpolation point planning algorithm. In the cutting experiment, the feed rate is 0.5 μm/r, the spindle speed is 1200 r/min, the cutting depth is 3 μm, and the diamond tool nose radius is 100 μm. The rake angle of diamond tool is 0° and the flank angle is 10°. In this experiment, the distances between the vibration measurement position and the workpiece rotation center are 10 mm, 30 mm and 50 mm respectively. See Table 6 for the measurement results. The surface of revolution machined in this experiment is a paraboloid, and its equation is $y=ax^{2}\;(a=1,2,4)$. As can be seen from Figure 20, compared with the equal residual height interpolation algorithm (ERH), the PSD value of machine tool vibration in the machining process of the adaptive interpolation algorithm (AIG) is obviously reduced, which shows that the adaptive interpolation algorithm can better suppress the vibration of the machine tool system. In addition, by comparing the data in Table 6, it can be found that the number of vibration frequencies in the machining process of adaptive interpolation algorithm is also significantly reduced, and it can also be seen from the blue highlighted data in the table that the vibration frequency bifurcation phenomenon is also suppressed. Moreover, the processing time of the two interpolation point planning algorithms is almost the same, which proves that the processing efficiency of the adaptive algorithm is not inferior to that of the traditional interpolation algorithm.

### 5.2. Reflectivity Verification

#### 5.2.1. Spherical Surface

**Figure 21:**
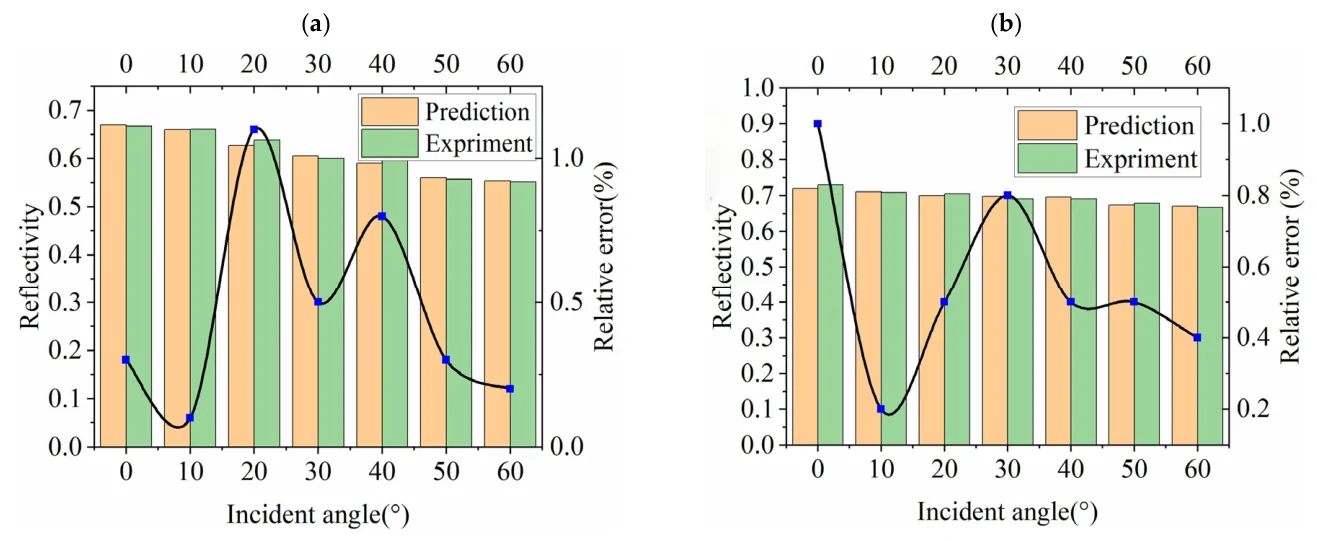
Absolute errors of theoretical and measured reflectance values in different bands: (**a**) visible light; (**b**) near-infrared (NIR).

By measuring the spherical machined surface, the reflectivity changes at different wavelengths are obtained as shown in Figure 21. The machining parameters in Figure 21 are the feed rate amount of 2 μm/r, the cutting depth of 2 μm, the spindle speed of 1200 r/min, and the tool nose radius of 100 μm; the incident ray wavelength is selected from 500 nm and 1200 nm. As can be seen from the figure, with the change in wavelength, the above theoretical analysis can well predict the change in reflectivity with vibration mode.

**Figure 22:**
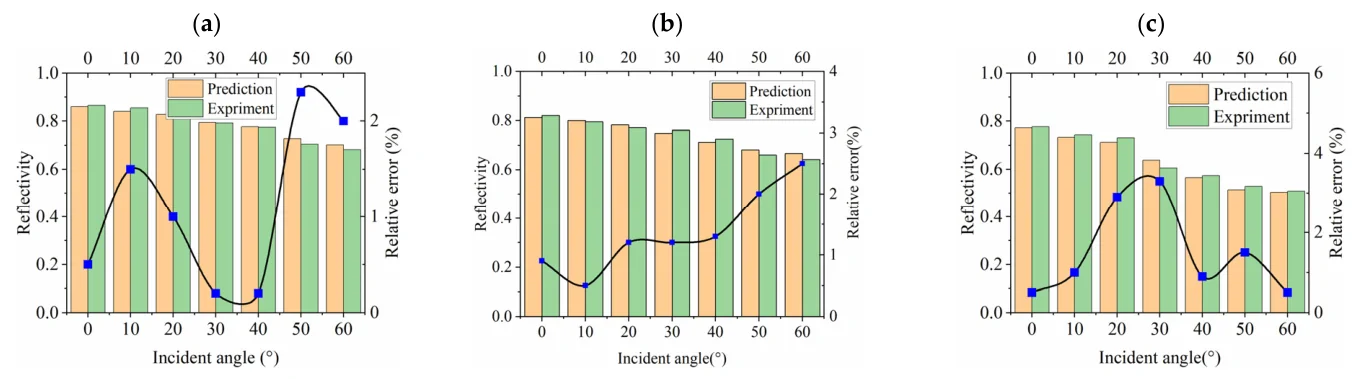
Absolute errors of theoretical and measured values of reflectivity under different calibers: (**a**) 2 mm; (**b**) 20 mm; (**c**) 50 mm.

The machining parameters in Figure 22 are set as the feed rate of 2 μm/r, the cutting depth of 2 μm, the spindle speed of 1200 r/min, the tool nose radius of 100 μm, and the incident ray wavelength of 500 nm is selected. Through measurement, it is obtained that the reflectivity changes under different diameters (2 mm, 20 mm and 50 mm) are shown in Figure 22. From the figure, the absolute error between the theoretical value and the experimental value is kept within 5%, indicating the accuracy of the theoretical model. As can be seen from the figure, with the increase in radial dimension, the reflectivity decreases gradually, which is the same as the change law of relative vibration of tool–workpiece. With the increase of radial dimension of workpiece, the cutting torque increases gradually, which makes the relative vibration of tool–workpiece more intense, and finally leads to more complex surface topography. According to the 3D topography model and reflectivity model, it can be known that the increase in P-V value of surface roughness leads to the decrease in reflectivity.

As shown in Table 7, for the comparison of reflectivity measurement results and simulation results under different diameters of machined spherical surfaces, the machining parameters in the table are set as feed rate of 1 μm/r, cutting depth of 2 μm, spindle speed of 1200 r/min, tool nose radius of 100 μm, and incident light wavelength of 500 nm. As can be seen from Table 7, with the decrease in spherical radius, the reflectivity gradually decreases under the same vector height, which can be explained by the author’s previous research results: With the decrease in spherical radius, the slope of workpiece surface increases, and with the increase in slope, the actual cutting depth increases, which eventually leads to the gradual intensification of vibration. According to the above reflectivity calculation model, with the intensification of vibration, the reflectivity decreases correspondingly.

#### 5.2.2. Revolution Paraboloid

**Figure 23:**
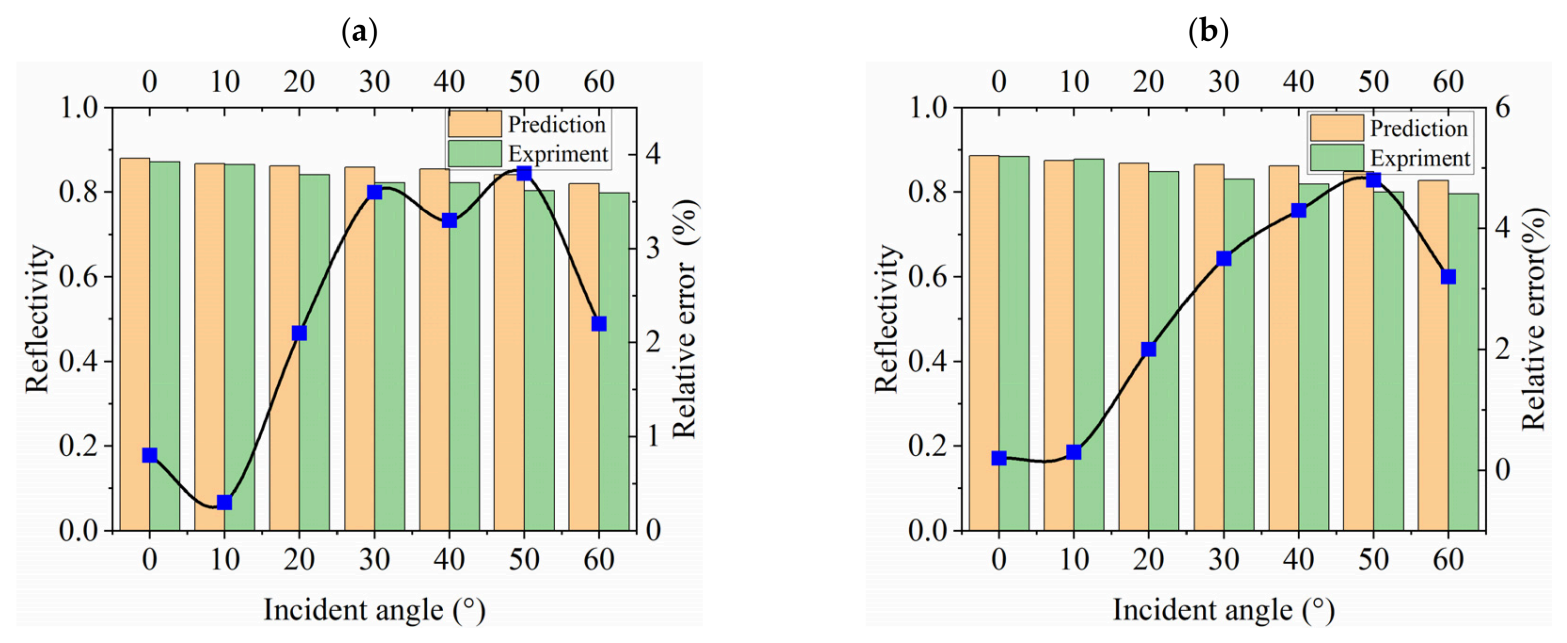
Absolute errors of theoretical and measured reflectance values in different bands: (**a**) visible light; (**b**) near-infrared (NIR).

Consistent with spherical surface machining, the machining parameters in this section are feed rate of 2 μm/r, cutting depth of 2 μm, spindle speed of 1200 r/min, tool nose radius of 100 μm, and incident light wavelengths of 500 nm and 1200 nm. Through measurement, it is found that the reflectivity changes at different wavelengths are shown in Figure 23. It can be seen from the figure that the above theoretical analysis can well predict the change trend of reflectivity with vibration modes. According to the change trend of reflectivity in the figure, it can be seen that the reflectivity decreases with the increase in incident angle and the influence of shadowing effect in any wavelength band, which is consistent with the conclusion obtained by our theoretical model, indicating that the influence of shadowing effect on surface reflectivity cannot be ignored.

**Figure 24:**
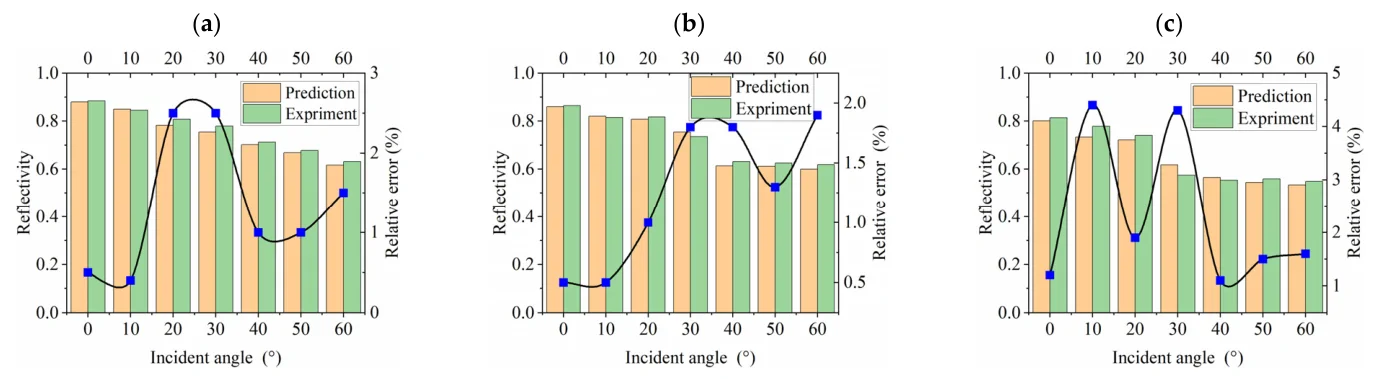
Absolute errors of theoretical and measured values of reflectivity under different calibers: (**a**) 2 mm; (**b**) 20 mm; (**c**) 50 mm.

The experimental processing parameters are set as feed rate of 2 μm/r, cutting depth of 2 μm, spindle speed of 1200 r/min, tool nose radius of 100 μm and incident light wavelength of 500 nm. Through measurement, the trend of reflectivity variation with different apertures is shown in Figure 24. From the figure, the absolute error between theoretical value and experimental value is kept within 5%, which shows the accuracy of the theoretical model. As can be seen from the figure, with the increase in aspheric machining caliber size, the reflectivity gradually decreases, which is the same as the change law of tool–workpiece relative vibration. With the increase in aspheric machining caliber, the cutting torque gradually increases, which makes the relative vibration between tool and workpiece more intense, and finally leads to more complex surface topography. The surface roughness P-V value increases from 18 nm to 46 nm. According to the three-dimensional topography model and reflectivity model, it can be seen that the surface roughness P-V value increases, which leads to the decrease in reflectivity.

As shown in Table 8, the reflectivity measurement results and simulation results under different machined aspheric equations are compared. The processing parameters in the table are a feed rate of 1 μm/r, a cutting depth of 2 μm, a spindle speed of 1200 r/min, a tool nose radius of 100 μm, and an incident light wavelength of 500 nm. As can be seen from Table 8, the equation of paraboloid is set as $z=ax^{2}$. With the increase in aspheric index *a*, the reflectivity gradually decreases under the same vector height. This phenomenon can be explained by the author’s previous research results: With the increase in the index, the slope of the workpiece surface increases, which makes the vibration gradually intensify. According to the above reflectivity calculation model, with the intensification of vibration, the reflectivity will decrease correspondingly.

### 5.3. Verification of the Effectiveness of Interpolation Planning Method

#### 5.3.1. Spherical Surface

**Figure 25:**
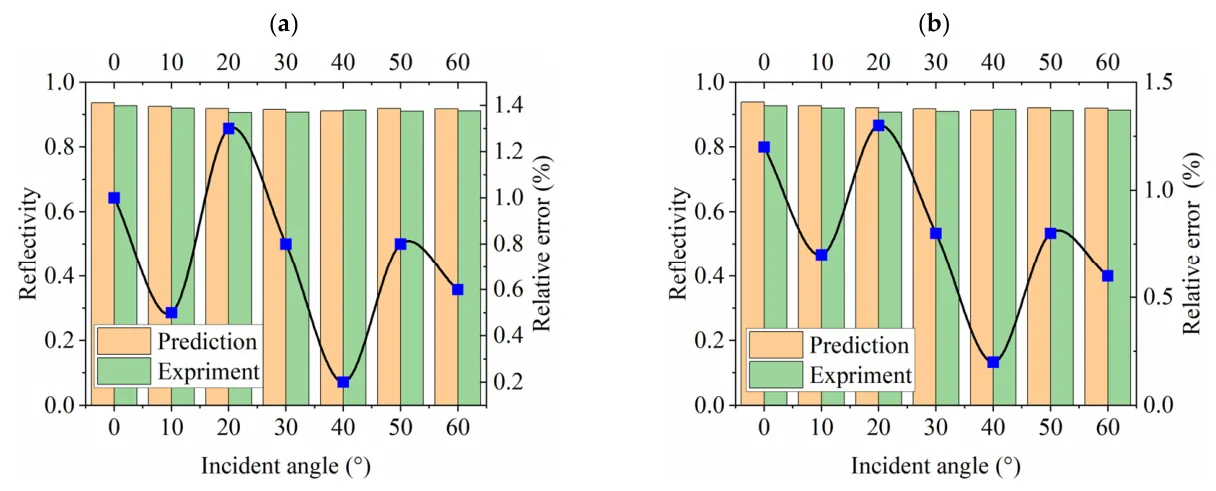
Absolute error of theoretical and measured reflectivity of spherical mirror at different wavelengths: (**a**) visible light; (**b**) near-infrared (NIR).

In Figure 25, the machining parameters are set as feed rate of 0.5 μm/r, cutting depth of 2 μm, spindle speed of 1200 r/min, tool nose radius of 100 μm, and incident light wavelengths of 500 nm and 1200 nm. By comparing the simulation results with the experimental measurement results, as shown in Figure 25, the absolute error is kept within 2%, which shows the accuracy of the simulation model calculation. By measuring the surface reflectivity of the spherical mirror, it can be seen that by using the new interpolation point planning algorithm, the mirror light reflectivity of the machined surface has been obviously improved, and the reflectivity can reach 93.6% (which has reached the ideal reflectivity). It shows that the interpolation point planning proposed in this paper can well restrain the relative vibration between the cutter and the tool when machining the spherical surface, and the surface roughness has also been greatly improved, so that the surface reflectivity has been greatly improved. As can be seen from Figure 25, even if the incident angle gradually increases, the reflectivity can still remain above 91%, indicating that the new interpolation method can effectively reduce the P-V value of the surface roughness and effectively restrain the influence of shadowing effect on the reflectivity.

**Figure 26:**
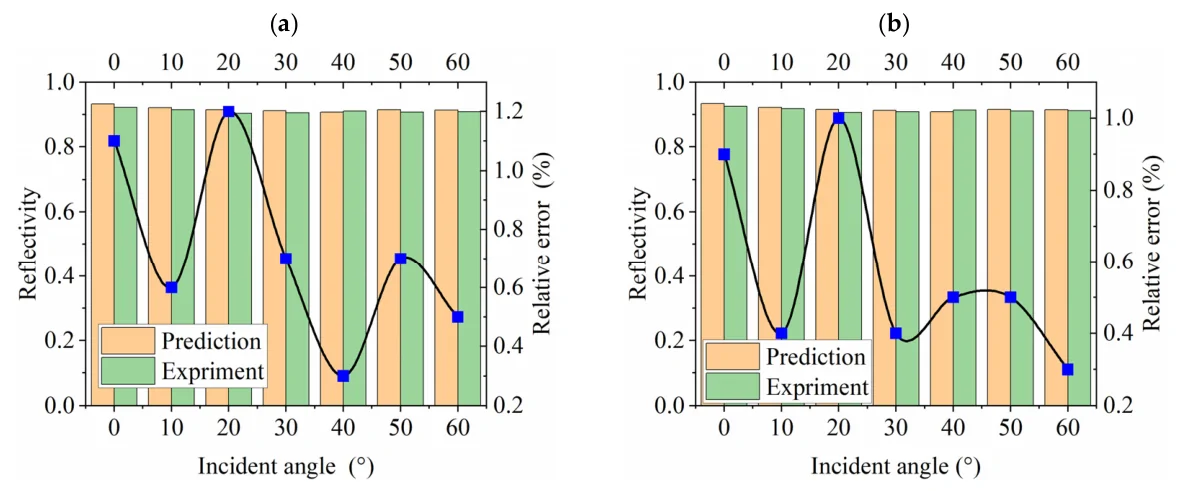
Absolute errors of theoretical and measured reflectivity values at different calibers after improving the interpolation point position: (**a**) 2 mm; (**b**) 50 mm.

**Figure 27:**
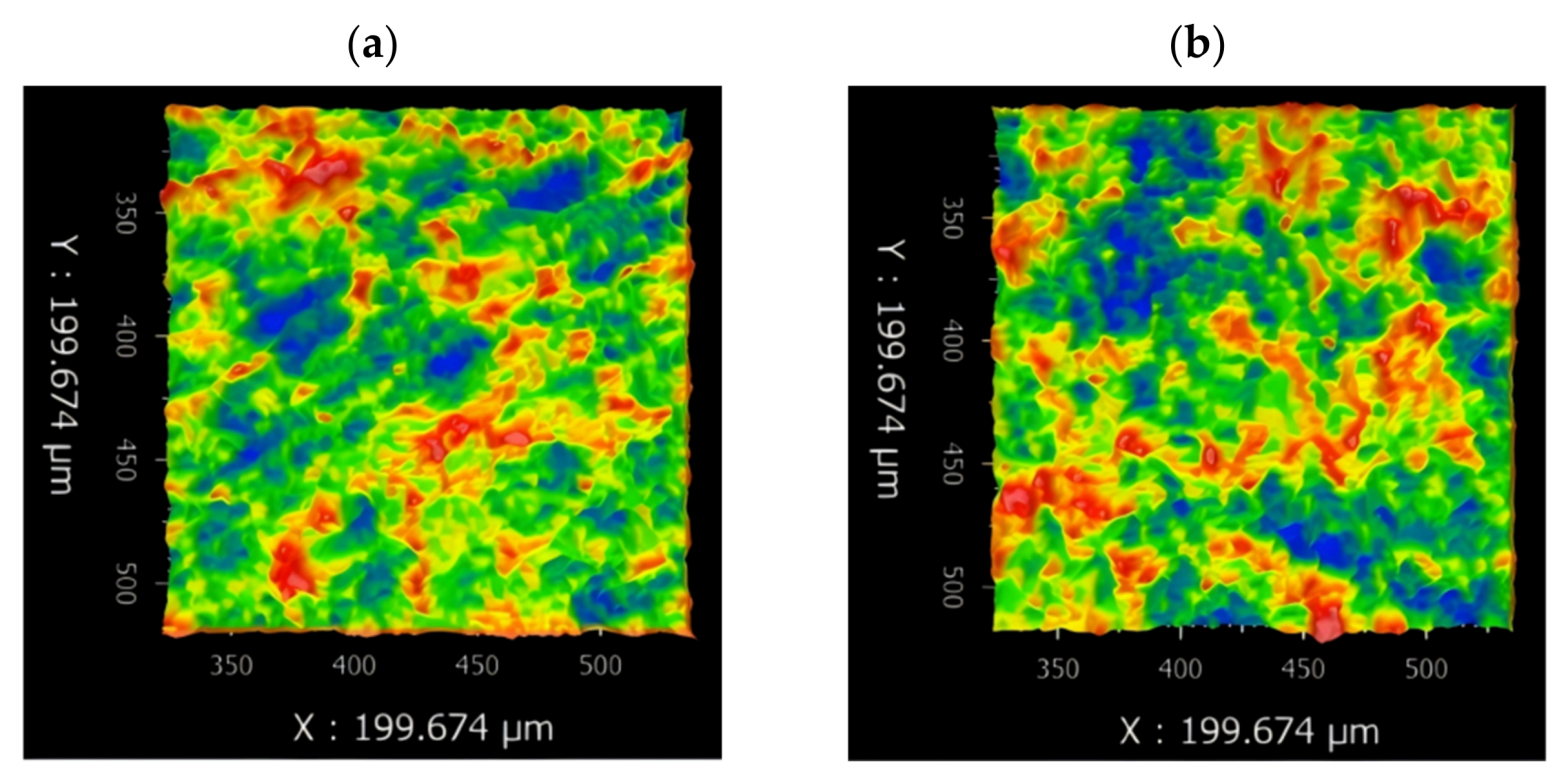
Surface topography of parabolic mirror at different cutting positions processed by adaptive interpolation algorithm: (**a**) 2 mm (P-V value = 12.2 nm); (**b**) 50 mm (P-V value = 11.1 nm).

The processing parameters are set as feed rate of 0.5 μm/r, cutting depth of 2 μm, spindle speed of 1200 r/min, tool nose radius of 100 μm, and incident light wavelength of 500 nm. Through measurement, the reflectivity changes of (2 mm and 50 mm) with different calibers are shown in Figure 26. From the figure, the absolute error between theoretical value and experimental value is kept within 5%, which shows the accuracy of the theoretical model. By measuring the reflectivity of different calibers on the surface of a spherical mirror, it can be seen that by using the new interpolation point planning algorithm, the reflectivity of the processed surface is obviously improved, and the reflectivity can reach 93.6% (which has reached the ideal reflectivity). Even if the caliber of the mirror is increased, the reflectivity can reach more than 91%. It shows that the interpolation point planning proposed in this paper can well restrain the relative vibration between tool and workpiece when machining spherical surface, and make the surface roughness greatly improved, thus making the surface reflectivity greatly improved. Even if the incident angle increases gradually, the reflectivity can still be kept at a high value, which shows that the new interpolation method can effectively restrain the influence of shadowing effect on reflectivity. As evidence, the results shown in Figure 27 indicate that the P-V value of the surface microstructure of the spherical reflector processed by the adaptive interpolation algorithm can reach within 12.5 nm.

**Figure 28:**
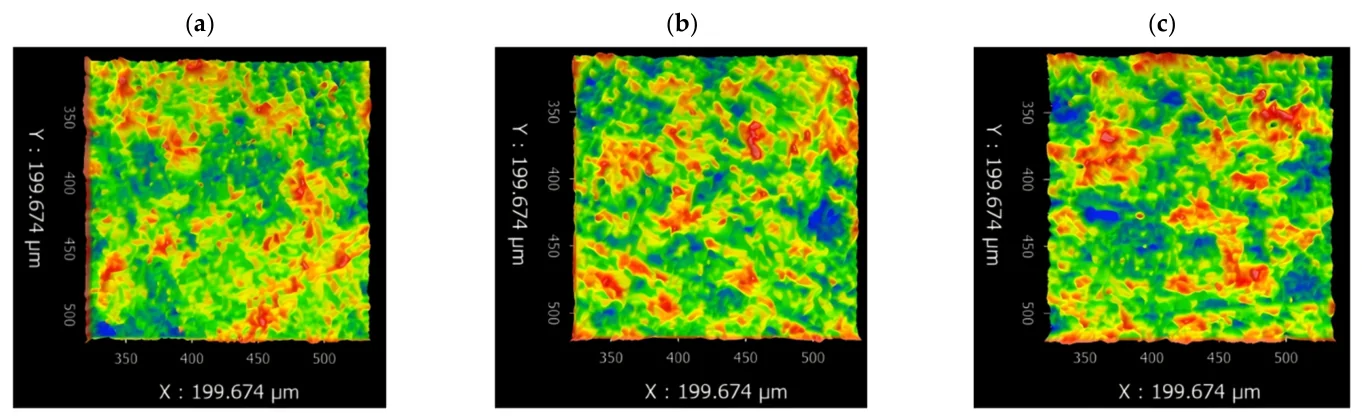
Machined surface of spherical mirrors with different radii processed by adaptive interpolation algorithm: (**a**) 30 mm (P-V value = 12.7 nm); (**b**) 40 mm (P-V value = 12.5 nm); (**c**) 50 mm (P-V value = 13.1 nm).

As shown in Table 9, for the comparison of reflectivity measurement results and simulation results under different diameters of machined spherical surfaces, the machining parameters in the table are set as feed rate of 1 μm/r, cutting depth of 2 μm, spindle speed of 1200 r/min, tool nose radius of 100 μm, and incident light wavelength of 500 nm. As can be seen from Table 9, by adopting the improved interpolation point planning algorithm, with the decrease in spherical radius, the reflectivity is always above 90% under the same vector height. This phenomenon shows that the vibration is well suppressed and the reflectivity will increase correspondingly. The above experimental observation results once again strongly prove that the adaptive interpolation algorithm can effectively reduce the P-V value of the processed surface of the parabolic reflector (see Figure 28 for supporting data), thereby significantly improving the reflectivity and stability of the parabolic reflector surface.

#### 5.3.2. Revolution Paraboloid

**Figure 29:**
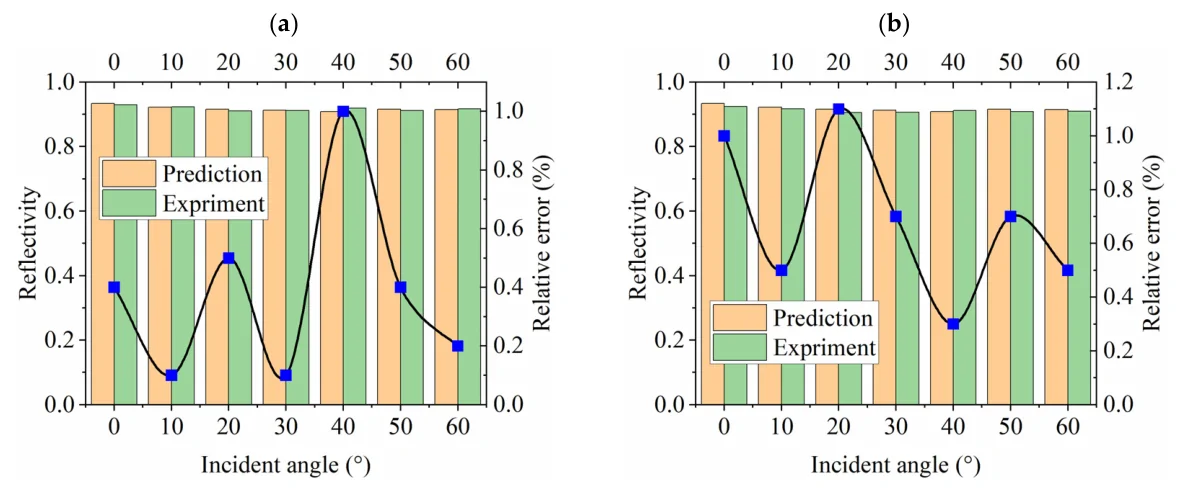
Absolute error of theoretical and measured reflectivity of parabolic mirror at different wavelengths: (**a**) Visible light; (**b**) near-infrared (NIR).

In Figure 29, the machining parameters are set as feed rate of 0.5 μm/r, cutting depth of 2 μm, spindle speed of 1200 r/min, tool nose radius of 100 μm, and incident light wavelengths of 500 nm and 1200 nm. Through the measurement of the surface reflectivity of the parabolic mirror, as shown in Figure 29, the absolute error between the experimental value and the theoretical value is kept within 2%. As can be seen from the figure, the mirror light reflectivity of the surface processed by the new interpolation point planning algorithm has been obviously improved, reaching the ideal reflectivity (93.6%) when the angle of incidence is 0 degrees, and the surface roughness has also been greatly improved. It shows that the interpolation point planning proposed in this paper can well restrain the relative vibration between tool and workpiece when processing aspheric surfaces, and then improve the reflectivity of the surface. Moreover, the surface shadowing effect of the mirror processed by the new interpolation algorithm is weakened, so that it can achieve the ideal reflectivity at any incident angle.

**Figure 30:**
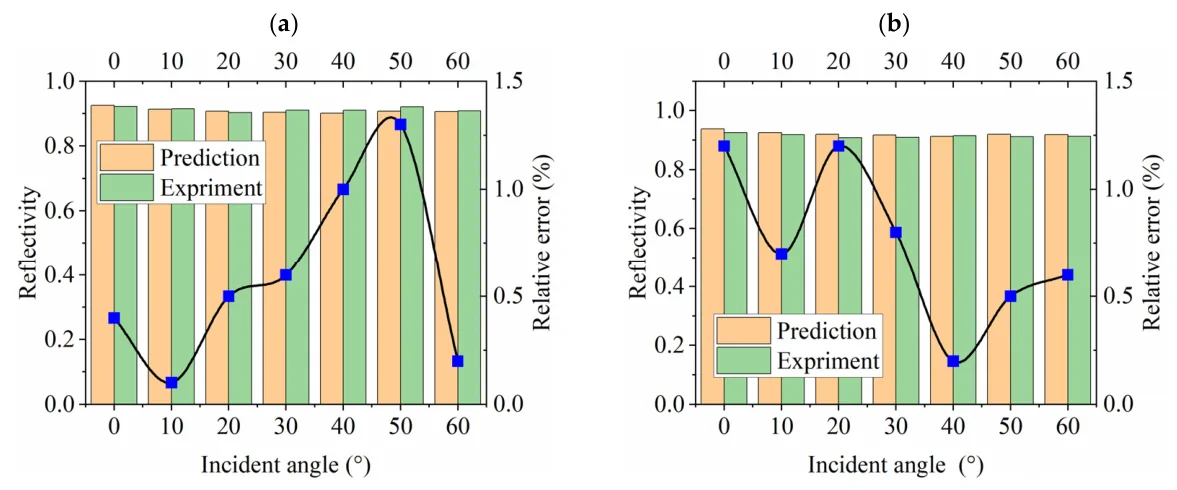
Absolute errors of theoretical and measured reflectivity values at different calibers after improving the interpolation point position: (**a**) 2 mm; (**b**) 50 mm.

**Figure 31:**
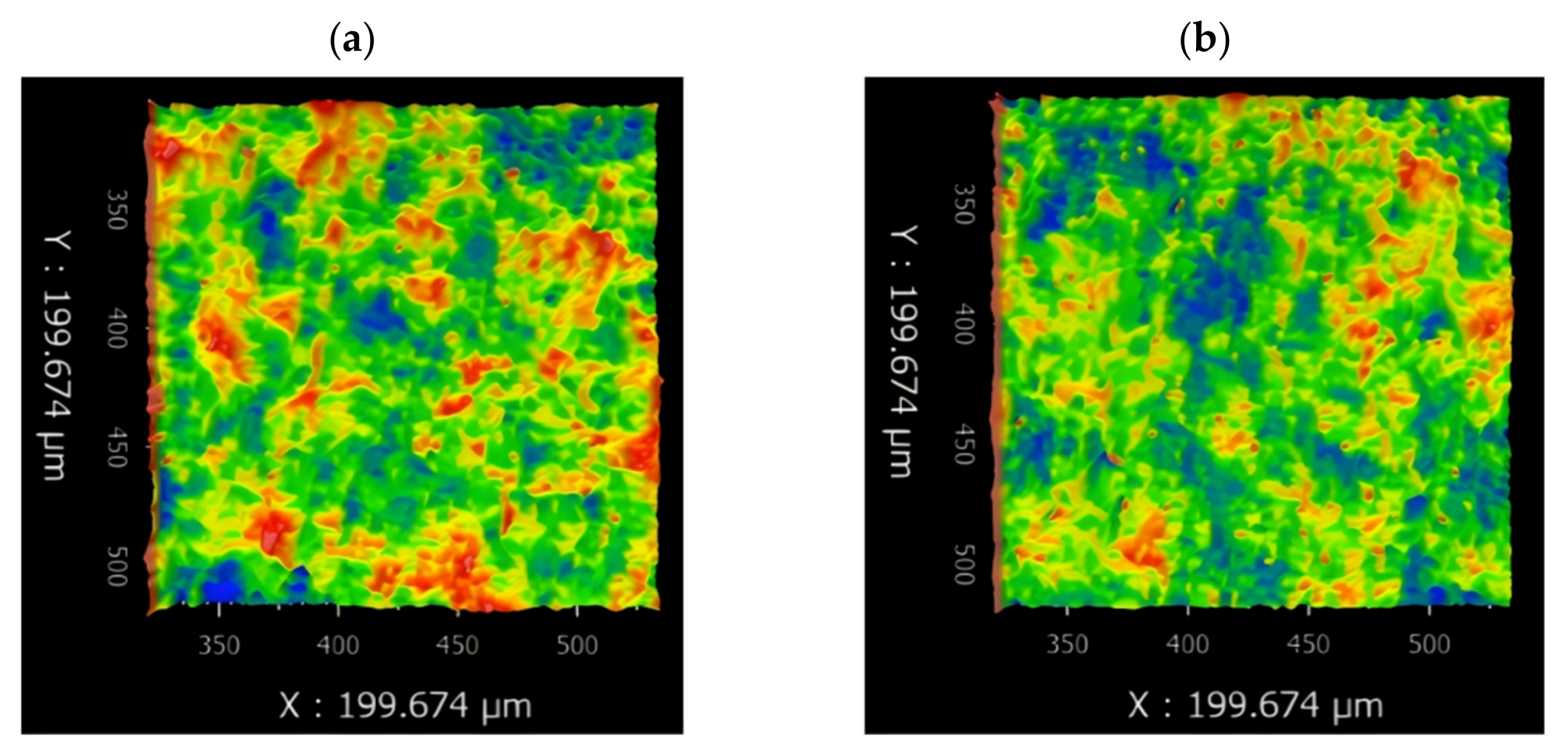
Surface topography of parabolic mirror at different cutting positions processed by adaptive interpolation algorithm: (**a**) 2 mm (P-V value = 13.3 nm); (**b**) 50 mm (P-V value = 12.7 nm).

In Figure 30, the machining parameters are set as feed rate of 0.5 μm/r, cutting depth of 2 μm, spindle speed of 1200 r/min, tool nose radius of 100 μm, and incident light wavelength of 500 nm. Through measurement, the reflectivity changes of (2 mm and 50 mm) with different calibers are shown in Figure 30. From the figure, the absolute error between theoretical value and experimental value is kept within 2%, which shows the accuracy of the theoretical model. By measuring the reflectivity of different calibers on the surface of the parabolic mirror of revolution, it can be seen that by using the new interpolation point planning algorithm, the reflectivity of the machined surface is obviously improved, and the reflectivity can reach 93.6% (reaching the ideal reflectivity). Even if the caliber of the mirror is increased, the reflectivity can reach more than 91%, which shows that the interpolation point planning proposed in this paper can well inhibit the relative vibration between the tool and the workpiece when machining the spherical surface. From the figure, the reflectivity can still be kept above 90% even if the incident angle gradually increases, which shows that the influence of shadowing effect on reflectivity is effectively suppressed. Figure 31 shows the surface microstructure and corresponding P-V values at different cutting positions measured by a white light interferometer. From the figure, it can be seen that even though the geometric shape of the workpiece surface is more complex than that of a standard spherical surface, the P-V value of the parabolic mirror machined surface can still reach around 13 nm.

**Figure 32:**
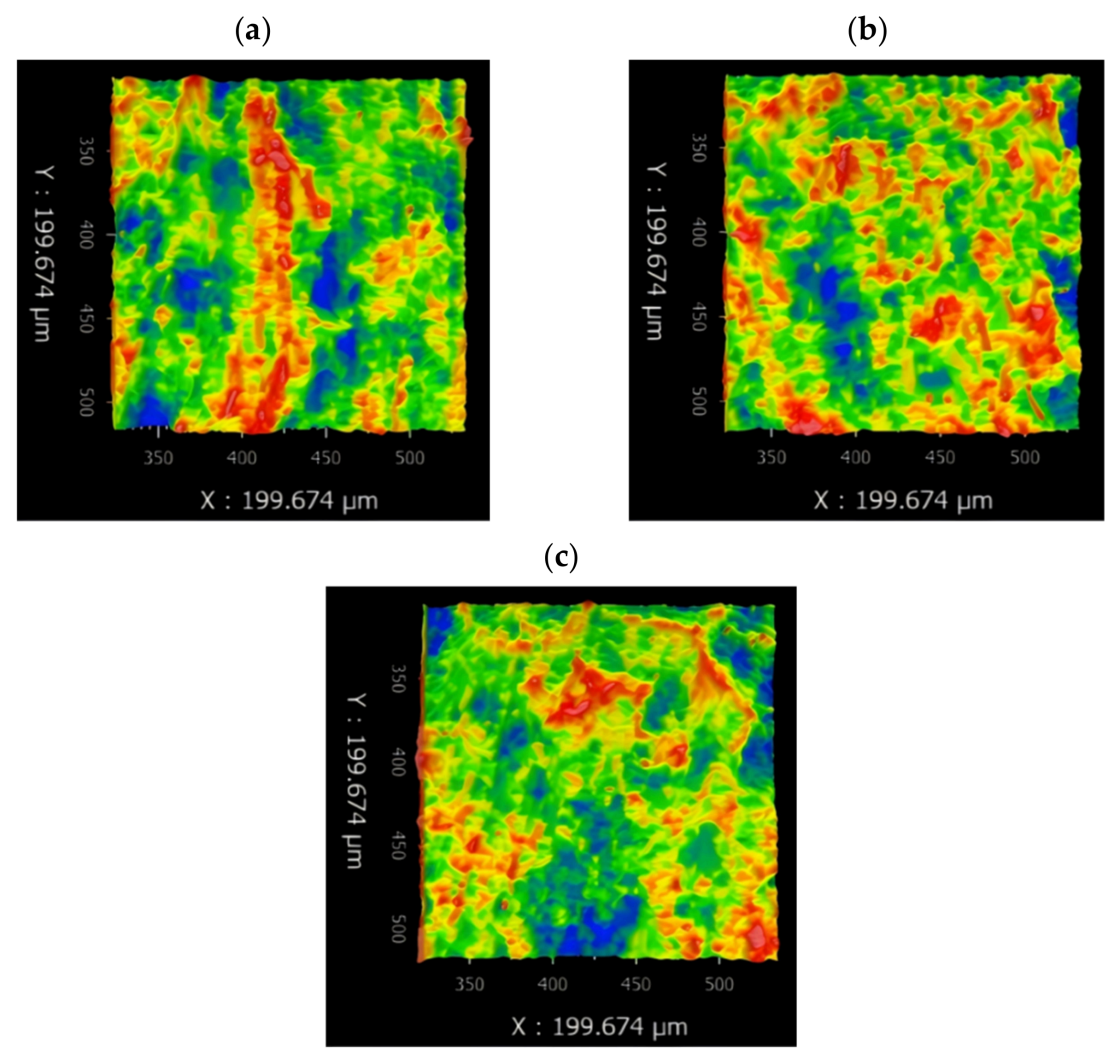
Surface morphology of parabolic mirrors with different shapes processed by adaptive interpolation algorithm: (**a**) a = 1 (P-V value = 13.2 nm); (**b**) a = 2 (P-V value = 13.8 nm); (**c**) a = 4 (P-V value = 13.0 nm).

As shown in Table 10, for the comparison between the reflectivity measurement results and simulation results under parabolic index *a*, the processing parameters in the table are set as feed rate of 1 μm/r, cutting depth of 2 μm, spindle speed of 1200 r/min, tool nose radius of 100 μm, and incident light wavelength of 500 nm. As can be seen from Table 10, by adopting the improved interpolation point planning algorithm, with the increase in the parabolic index *a*, the reflectivity is always kept above 90% under the same vector height. This phenomenon shows that the vibration is well suppressed and the reflectivity will increase accordingly. The above experimental observation results once again strongly prove that the adaptive interpolation algorithm can effectively reduce the P-V value of the processed surface of the parabolic reflector (see Figure 32 for supporting data), thereby significantly improving the reflectivity and stability of the parabolic reflector surface.

## 6. Conclusions

In this paper, the influence of vibration modes on reflectivity is systematically analyzed, and the vibration is suppressed by different means, the peak-valley value of workpiece surface roughness is reduced, and the reflectivity of machined surface is improved. According to the above results and analysis, the following conclusions can be drawn:

The reflectivity of ultra-precision turning surface is calculated by rigorous coupled wave method, and the influence of aberration on reflectivity is considered in combination with the specific structural parameters of spherical/aspherical surface. The relationship between reflectivity and surface morphology is strictly calculated. According to the influence of different vibration frequencies on the surface topography, the influence of shadowing effect on reflectivity in different wavelength bands is studied. It is concluded that the reflectivity decreases gradually with the increase in incident angle, whether in ultraviolet, visible light or near-infrared bands, and the influence of shadowing effect on the reflectivity in ultraviolet band is more serious.

The established multi-body dynamics equations are used to predict the effects of different factors on the vibration frequency bifurcation and the variation in vibration amplitude of the ultra-precision machine tool. Through calculation results, it was found that when cutting spherical and non spherical mirrors, the phenomenon of vibration frequency bifurcation often occurs as the distance between the cutting position and the center of rotation of the workpiece increases.

In this paper, a new adaptive interpolation point planning algorithm is obtained by studying the limit cutting depth which causes the vibration bifurcation phenomenon, and the interpolation point position on the meridian of the workpiece is planned. When the interpolation point optimization algorithm is adopted, the relative vibration between the tool and the workpiece is obviously weakened, resulting in the reduction in the P-V value of the machined surface roughness. The decrease in roughness values greatly reduces the diffraction phenomenon on the surface of the mirror and increases the reflectance of the mirror surface. Even with the gradual increase in incident angle, the specular reflectivity of uncoated bare spherical/aspherical mirror surface can reach more than 90%, which shows that with the decrease in vibration, the shadowing effect is well suppressed. The specific program of the interpolation algorithm in this article is detailed in the attachment (Supplementary Materials).

## Figures and Tables

**Table 1:** Variation in shadowing effect coefficient with incident angle.

Incident Angle (°)	Parameter *a*(*n*)	Parameter *b*(*n*)
0	2.54	1.92
10	2.6	2.08
20	2.67	2.41

**Table 2:** Variation in vibration frequency and amplitude with equal-feed interpolation algorithm (The part filled with colors in the figure represents the location where the vibration frequency bifurcation phenomenon occurs).

*r_n_* (mm)	Frequency (Hz)/Amplitude (nm)									
5	135/2.3	273/6.2	794.4/ 8.0		1050/3.2	1200/2.5		2200/4.5	4536/3.4	6750/2.0
10	135/2.4	273.4/7.6	795/ 12.5		1050/4.5	1200/4.8		2200/6.8	4524.7/4.8	6750/3.8
15	135/6.7	273.6/8.4	795.4/ 10.9		1050/10.5	1139/6.7	1245/5.4	2200/9.7	4526/4.7	6750/3.9
40	135/7.6	275.5/8.5	792.5/14.9		1050/14.8	1047/15.8	1391/6.7	2200/10.8	4517/5.9	6750/3.8
45	135/7.9	275.8/8.0	757.3/15.2	946.3/7.2	1050/18.7	1023/21.9	1423/8.9	2200/13.8	4533/6.8	6750/4.4
75	135/8.9	277.9/9.7	558.6/18.7	1104.7/8.5	1050/22.8	874/30.9	1640/9.2	2200/14.9	4568/7.9	6750/4.8

**Table 3:** Variation in vibration frequency and amplitude with equal-residual-height interpolation algorithm (The part filled with colors in the figure represents the location where the vibration frequency bifurcation phenomenon occurs).

*r_n_* (mm)	Frequency (Hz)/Amplitude (nm)									
5	135/2.1	273/4.5	794.4/3.2	1050/3.0	1200/3.7		2200/3.1	4536/2.2		6750/2.2
10	135/2.0	273.4/4.7	795/3.3	1050/3.3	1200/4.8		2200/3.7	4524.7/5.8		6750/3.5
25	135/4.4	274.4/7.2	796/5.9	1050/4.8	1105/12.8	1315/4.8	2200/7.2	4513/7.9		6750/4.6
60	135/6.5	276.9/12.5	795/14.5	1050/5.8	963/16.9	1527/6.7	2200/14.8	4537/16.7		6750/6.2
65	135/6.3	277.2/14.3	797.9/17.2	1050/6.7	942/18.1	1563/7.5	2200/19.2	4399/21.2	4623/7.8	6750/6.1
75	135/7.8	277.9/15.4	798.6/19.2	1050/7.2	874/20.0	1640/7.8	2200/22.7	4358/22.8	4668/7.1	6750/6.6

**Table 4:** Sensitivity gradient modulus and sensitivity factor of the concerned variables.

Influencing Factors	Modes of Sensitive Gradients *A*	Sensitivity Factor $\lambda_{k}$
Depth of cut	0.0937	30%
Feed rate	0.0254	8%
Spindle speed	0.0633	20%
Workpiece surface slope	0.0854	27%
Radii of tool nose	0.0458	15%

**Table 5:** Comparison of vibration modes of workpieces with different calibers.

	Frequency	1050 Hz	2400 Hz	3200 Hz
Caliber	Amplitude			
2 mm		1.0 nm	0.8 nm	1.2 nm
20 mm		1.5 nm	1.1 nm	1.9 nm
50 mm		0.9 nm	1.2 nm	1.4 nm

**Table 6:** Effects of adaptive interpolation point planning algorithm and equal residual height interpolation point planning method on machine tool system vibration.

Vibration Frequency (Hz)										Processing Time (s)
*r_n_* = 10 mm	ERH	135	273.6	1050	1200	2200	4526	4587	6750	1178
	AIG			1050		2405	4500		6649	1189
*r_n_* = 30 mm	ERH	135	273.6	1050	1239	1245	4526			3157
	AIG				1200	3206	4517			3292
*r_n_* = 50 mm	ERH	135	277.9	874	1212	1298	1640	4358	4668	5438
	AIG			850		4672				5513

**Table 7:** Absolute errors of theoretical and measured reflectivity under different spherical diameters.

Spherical Radius (mm)	Reflectance Calculated Value	Reflectance Measurement Value	Absolute Errors
300	0.885	0.882	0.3%
400	0.892	0.887	0.5%
500	0.865	0.869	0.4%

**Table 8:** Absolute errors of theoretical and measured values of reflectivity under different *a* values.

Index *a*	Reflectance Calculated Value	Reflectance Measurement Value	Absolute Error
1	0.89	0.885	0.5%
2	0.88	0.882	0.2%
4	0.892	0.894	0.2%

**Table 9:** Absolute errors of theoretical and measured reflectivity under different spherical diameters.

Spherical Radius (mm)	Reflectance Calculated Value	Reflectance Measurement Value	Absolute Errors
300	0.929	0.931	0.2%
400	0.925	0.92	0.5%
500	0.924	0.928	0.4%

**Table 10:** Absolute errors of theoretical and measured values of reflectivity under different *a* values.

Index *a*	Reflectance Calculated Value	Reflectance Measurement Value	Absolute Errors
1	0.929	0.931	0.2%
2	0.925	0.92	0.5%
4	0.924	0.928	0.4%

## Data Availability

The authors declare that all data supporting the findings of this study are available within the article.

## Supplementary Materials

The following supporting information can be downloaded at: https://www.mdpi.com/article/10.3390/mi17080938/s1.
