# Supplementary material for: An Investigation into the Planning of Cutting Interpolation Point Positions to Improve the Surface Quality of Satellite Laser Communication Reflectors
Source: Micromachines (Basel). 2026 Aug 6;17(8):938. doi: 10.3390/mi17080938 (PMC13515171; doi:10.3390/mi17080938)
Supplement: Supplementary file 1 [file micromachines-17-00938-s001.zip › micromachines-4453011-supplementary.pdf]

```
import numpy as np
import matplotlib.pyplot as plt

class AdaptiveInterpolationAlgorithm:
    def __init__(self, workpiece_radius, initial_feed_rate, min_feed_rate=0.1):
        """
        Initialize the adaptive interpolation algorithm.
        :param workpiece_radius: Workpiece radius R (mm).
        :param initial_feed_rate: Initial feed rate fi (um/rev) - suggested as 0.5 um/r in Section
3.3.2.
        :param min_feed_rate: Minimum allowable feed rate (um/rev) due to machine limits.
        """
        self.R = workpiece_radius
        self.fi = initial_feed_rate
        self.f_min = min_feed_rate

        # ---------------------------------------------------------
        # REVIEWER COMMENT CLARIFICATION (C1):
        # The following dictionary `max_depth_slope_curve` is a SIMPLIFIED PROXY
        # used for algorithm demonstration. It is NOT a direct, exact 1:1 mapping
        # of the empirical data shown in Figure 17 of the manuscript.
        # Instead, it represents the physical approximation of the trend observed
        # in Figure 17: as the surface slope increases, the limit cutting depth
        # (threshold for vibration frequency bifurcation) decreases.
        # In actual industrial application, this should be replaced with the exact
        # fitted polynomial equation or the raw experimental dataset from Figure 17.
        # ---------------------------------------------------------
        self.max_depth_slope_curve = {
            0.1: 4.7,    # Slope 0.1 -> Limit depth 4.7 um (4700 nm)
            0.2: 4.0,    # Slope 0.2 -> Limit depth 4.0 um
            0.3: 3.9,
            0.4: 3.7,
            0.5: 3.5
        }

    def get_surface_slope(self, x):
        """
        Calculate the surface slope (dz/dx) of the workpiece at radial position x.
        """
        # Physical constant approximation:
        # Assumed radius of curvature for a parabolic mirror (R_curve = 100 mm).
        # For a standard parabolic equation z = x^2 / (2*R_curve),
        # the derivative is dz/dx = x / R_curve.
        # This is a geometric approximation used for the simulation.
```

```
        R_curve = 100.0
        slope = x / R_curve
        return abs(slope)

    def get_limit_cutting_depth(self, slope):
        """
        Obtain the limit cutting depth (in nm) based on the current slope.
        Uses linear interpolation on the simplified proxy curve.
        """
        slopes = np.array(list(self.max_depth_slope_curve.keys()))
        depths = np.array(list(self.max_depth_slope_curve.values()))

        # If the slope exceeds the defined range, use the minimum boundary value
        if slope >= slopes[-1]:
            return depths[-1]

        # Linear interpolation to approximate intermediate limit depths
        limit_depth = np.interp(slope, slopes, depths)
        return limit_depth

    def calculate_actual_cutting_depth(self, feed_rate, slope):
        """
        Estimate the actual depth of cut.
        """
        # Physical approximation:
        # This is a simplified kinematic model for demonstration purposes.
        # As discussed in Section 3.1.1 (e.g., Equation 20), the rigorous actual
        # depth of cut is a complex function involving the tool nose radius (RT),
        # feed rate, and geometric included angles.
        # Here, it is approximated proportionally based on feed rate and slope
        # to simulate the vibration risk triggering mechanism.
        # Multiplied by 1000 to convert units for consistent comparison (nm scale).
        return feed_rate * slope * 1000

    def generate_toolpath(self):
        """
        Execute the adaptive interpolation algorithm.
        This directly corresponds to the optimization flow chart shown in Figure 18.
        """
        x_positions = [0.0]          # List to store interpolation point positions
        feed_rates = [self.fi]       # List to store corresponding feed rates

        current_x = 0.0
        current_f = self.fi
```

```python
        print(f"{'Position(mm)':<15} | {'Slope':<10} | {'Limit Depth(nm)':<16} | {'Actual Depth(nm)':<16} | {'Feed Rate(um/r)':<16} | {'Status'}")
        print("-" * 95)

        while current_x < self.R:
            # 1. Predict the next interpolation point based on current feed rate
            next_x = current_x + current_f / 1000.0 # Convert um to mm

            if next_x > self.R:
                next_x = self.R

            # 2. Acquire current machining environment parameters
            slope = self.get_surface_slope(next_x)
            limit_depth = self.get_limit_cutting_depth(slope)

            # 3. Calculate actual cutting depth under current feed rate
            # In the manuscript, actual cutting depth acts as an indicator for dynamic force
            # and potential vibration frequency bifurcation.
            actual_depth = self.calculate_actual_cutting_depth(current_f, slope)

            # 4. Determine whether vibration frequency bifurcation has occurred
            # (i.e., check if actual depth exceeds the theoretical limit depth)
            if actual_depth > limit_depth:
                # Bifurcation risk detected.
                # Need to recalculate and reduce feed rate (f') as per Fig. 18.
                safety_factor = 0.9  # Physical constant: 10% safety margin applied during deceleration
                new_f = current_f * (limit_depth / actual_depth) * safety_factor

                if new_f < self.f_min:
                    new_f = self.f_min
                    status = "Hit Min Feed Rate"
                else:
                    status = "Adaptive Deceleration"

                current_f = new_f
                # Recalculate the actual depth with the newly optimized feed rate
                actual_depth = self.calculate_actual_cutting_depth(current_f, slope)
            else:
                status = "Normal Machining"

            # 5. Record the optimized data points
            x_positions.append(next_x)
```

```
            feed_rates.append(current_f)

            print(f"{next_x:<15.4f}    |    {slope:<10.4f}    |    {limit_depth:<16.2f}    |
{actual_depth:<16.2f} | {current_f:<16.4f} | {status}")

            current_x = next_x

        return x_positions, feed_rates

# --- Main Execution Block ---
if __name__ == "__main__":
    # Parameters setup referring to Manuscript Section 4.2 and 3.3.2
    # Workpiece radius = 50 mm, Initial feed rate = 0.5 um/r
    algo = AdaptiveInterpolationAlgorithm(workpiece_radius=50, initial_feed_rate=0.5)

    # Run the adaptive interpolation path planning
    x_path, f_path = algo.generate_toolpath()

    # Visualization of the results
    plt.figure(figsize=(10, 6))
    plt.plot(x_path, f_path, marker='.', linestyle='-', color='b')
    plt.title('Adaptive Interpolation: Feed Rate vs. Position')
    plt.xlabel('Position on Meridian (mm)')
    plt.ylabel('Feed Rate (um/rev)')
    plt.grid(True)
    plt.show()
```
